# Isolation and characterization of heavy metal tolerant microalgae from old mining areas of Saxony

**DOI:** 10.1038/s41598-025-32393-0

**Published:** 2026-01-05

**Authors:** Khongorzul Mungunkhuyag, Juliane Steingroewer, Thomas Walther, Felix Krujatz

**Affiliations:** 1 Chair of Bioprocess Engineering, Institute of Natural Materials Technology, Technische Universität Dresden, 01069 Dresden, Germany; 2 Department of Biology, School of Arts and Sciences, National University of Mongolia, Ulan-Bator, 14200 Mongolia; 3 biotopa gGmbH, 01454 Radeberg, Germany; 4 Professorship Automatic Control and System Dynamics, Technische Universität Chemnitz, Reichenhainer Straße 70, 09126 Chemnitz, Germany

**Keywords:** Microalgal isolates, Phylogenetic analysis, Heavy metal tolerance, Heavy metal removal, Biotechnology, Environmental sciences, Microbiology

## Abstract

Heavy metal contamination poses a major threat to ecosystems and human health, particularly in mining-impacted areas. Algal biosorption offers a promising, low-cost, and sustainable approach to mitigate this problem. In this study, five microalgal strains were bioprospected and isolated from abandoned mining sites and evaluated for their tolerance and ability to remove Cu, Cd, and Cr(VI) from aqueous solutions. Laboratory experiments were performed to assess heavy metal tolerance and biosorption efficiency under controlled conditions. The results demonstrated that isolates *Chlorella vulgaris* RG1-4 and *Tetradesmus obliquus* Ehr33-9 exhibited the highest tolerance and biosorption capacity for Cu, with removal efficiencies of 100.00 mg/g and 89.73 mg/g, respectively. The isolate *Lobochlamys segnis* Ehr31-1 showed the highest tolerance and biosorption capacity for Cd, reaching 93.17 mg/g. These findings highlight the potential of locally adapted microalgal strains as effective biosorbents for remediating heavy metal-contaminated water sources.

**Supplementary Information:**

The online version contains supplementary material available at 10.1038/s41598-025-32393-0.

## Introduction

 Heavy metals (HMs) and metalloids are elements with a density exceeding 5 g/cm^3^ that can be toxic to humans and other (micro)organisms, even at low part per billion (ppb) concentrations^1^. They can enter the environment through natural processes, such as soil erosion, forest fires, and volcanic eruptions, as well as through anthropogenic activities like mining, electroplating, and the use of pesticides, fertilizers, and certain consumer products^2^. If contaminated areas are not remediated, HMs can pollute surrounding water and soil, leading to human exposure via ingestion, inhalation, or dermal absorption^3^. Once inside the body, HMs can bind to biomolecules, disrupting biochemical pathways and impairing cellular functions. This can cause an ion imbalance and generate reactive oxygen species (ROS), potentially harming various organs and increasing cancer risk^2,4^. Among the HMs, nickel (Ni), cadmium (Cd), arsenic (As), hexavalent chromium (Cr(VI)), uranium (Ur), and the fission product strontium (Sr) are classified as Group 1 HMs by the International Agency for Research on Cancer (IARC), indicating their carcinogenic potential to humans^5^. 

When entering cells, Cd binds to sulfhydryl groups (-SH) in proteins, particularly amino acid cysteine, which can inhibit enzyme function and damage cellular structures like the nucleus, the endoplasmic reticulum, and mitochondria. This interference also affects DNA methylation, leading to epigenetic changes and potential health issues in humans, such as liver and kidney damage, bone mineralization loss (e.g., Itai-Itai disease), and cancer^6^. Copper is an essential element for biological functions, acting among others as a cofactor in various enzymes that are involved in energy production, iron metabolism and neurotransmitter synthesis. Thus, adequate copper intake is crucial for human health^7^. However, excessive copper exposure can cause liver and gastrointestinal damage^8^. Copper is even more toxic to microorganisms and aquatic organisms^9,10^. Its use in agriculture and as a food preservative should therefore be carefully managed. Otherwise, it can negatively impact the ecosystem by stunting the growth of aquatic organisms and altering the composition of microorganisms. Chromium exists in several oxidation states, with Cr(III) and Cr(VI) being the most stable, whereas Cr(VI) shows the highest toxicity. When Cr(VI) enters cells, it generates ROS, which can damage intracellular macromolecules such as lipids, proteins, and nucleic acids. This can ultimately lead to allergic reactions, damage to sperm and the male reproductive system, and an increased risk of cancer^11^.

Trace concentrations of HMs, measured in ppb can significantly impact human health and the environment. Regulatory frameworks, such as those established by the World Health Organization (WHO), set stringent limits for HMs in drinking water exemplarily shown for cadmium at 0.003 mg/L, arsenic at 0.01 mg/L, lead at 0.01 mg/L, chromium at 0.05 mg/L, and copper at 2 mg/L^12^. Higher limits may apply to industrial effluents and irrigation water^13,14^. HM concentrations near mining operations depend on the soil exposure to contaminated waters. For example, the Meca River in Spain, affected by acid mine drainage, showed an arsenic load at 0.13 mg/L, cadmium at 0.8 mg/L, copper at 9.2 mg/L, lead at 0.36 mg/L, and zinc at 4.09 mg/L^15^. In contrast, the Ib Valley Coalfield in India reported lower levels of these metals^16^. Industrial processes also contribute to HM pollution. Wastewater from an electroplating factory contained copper concentrations up to 15,560 mg/L, along with significant levels of nickel (245 mg/L) and zinc (505 mg/L). Additionally, the lead concentration in a wastewater from an industrial area in Delhi was found to be 326 mg/L while the cadmium concentration was found to be 0.9 mg/L^17^.

HMs can be removed from water using various methods categorized as chemical, physical, and biological techniques. Chemical and physical unit operations include adsorption, precipitation, membrane filtration, ion exchange, solvent extraction, electro-dialysis, neutralization, coagulation, and flocculation^18^. These methods often entail high costs for equipment and reagents, may not completely eliminate metals, and can cause secondary pollution requiring further treatment^19^. Their effectiveness also decreases at low metal concentrations (10–100 mg/L). In contrast, a biological treatment, or bioremediation, employs (micro)organisms such as plants, bacteria, yeast, fungi, and algae, showing a HM removal effectiveness even at low HM concentrations^20^. Bioremediation is cost-effective and environmentally friendly, although it can be time-consuming and may lack specificity in targeting various metal ions^19^.

Among them algae are a group of photosynthetic organisms which can grow without organic carbon sources and are effective in fixing carbon dioxide, making them valuable for reducing greenhouse gas emissions. Microalgae, in particular, are versatile microorganisms and can be cultivated in various technological systems^21^, demonstrating high tolerance to HMs as well. Microalgae exhibit higher HM biosorption capacities than conventional adsorbents, making them promising for environmental remediation. For instance, *Spirulina platensis* absorbs Cd at over 120 mg/g^22^, while *Chlorella vulgaris* absorbs 17.46 mg/g^23^, compared to lignite’s 40.46 mg/g^24^. For Cu, *Callithamnion corymbosum* showed an absorption capacity of 81.25 mg/g, and a biosorbent from agricultural waste revealed 63.37 mg/g^25^. *Chlorella vulgaris* ZBS1 absorbed 74.63 mg/g of Cr(VI)^26^, while woody-activated carbon can absorb 241.6 mg/g^27^.

Microalgae have developed different mechanisms to tolerate high concentrations of HM. These mechanisms are: adsorption at the cell surface via extracellular polymeric substance or cell wall components, intracellular accumulation of metals to certain metalloproteins and other chelators, compartmentalization, converting metals into less toxic forms, or an active efflux transport^28^.

While much research has focused on cultured algae, less attention has been given to bioprospected algae from HM-contaminated environments. Saxony, with its 800-year mining history^29^, may harbor a diversity of microalgae that are well adapted to such conditions and exhibit high absorption capacities. This study aimed to isolate HM-tolerant microalgal species from mining areas, characterize them through molecular analysis, evaluate their tolerance and growth in response to copper (Cu), cadmium (Cd), and chromium (Cr(VI)), and assess their capability for HM removal. The reason those HM were studied is that first, even small concentrations of those metals can be toxic to the environment and human health. Secondly, the concentrations of Cu and Cd were elevated in the specific sampling sites in the Erzgebirge^30^.

## Results and discussion

### Isolation and pre-screening of HM tolerant isolates

A total of 51 microalgae cultures were isolated from the collected samples, consisting of 44 green microalgae and 7 cyanobacterial strains. In a pre-screening process the 51 isolates were exposed to selected HMs to identify promising candidates for a deep physiological and HM tolerance analysis. As shown in Table 1, most of the isolates exhibited tolerance to As(V) at 100 mg/L and Pb at 20 mg/L. However, Cd, Cu, and Cr(VI) were highly toxic, particularly Cr(VI), with only few microalgae showing tolerance to these HMs.

**Table 1:**
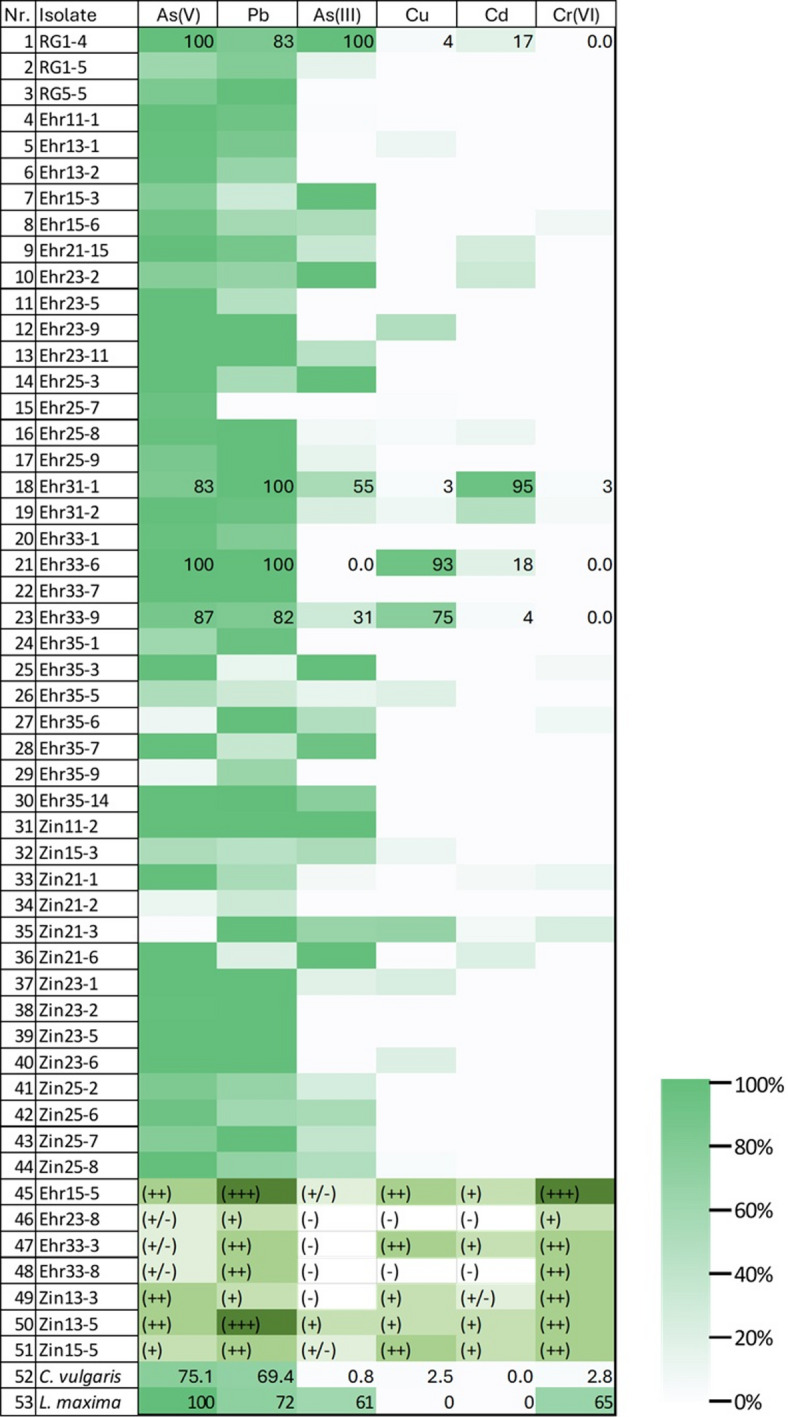
Algal tolerance to heavy metals. For green algae, the growth in the media containing heavy metals were compared to growth of the isolates in the normal medium (control) presented as percentage and the colour scale is given beside the table. For cyanobacteria, the growth was compared to its control and the coloration was measured by naked eye. The meaning of the marks: (+++) – growth is same as control, (++) - growth is slightly lower than control, (+) – growth is low, (+/-) – no growth, (-) – discoloration. *C. vulgaris* (*Chlorella vulgaris* SAG211-11b) and *L. maxima* (*Limnospira maxima* CCALA27) are reference strains for comparison.

**Fig. 1:**
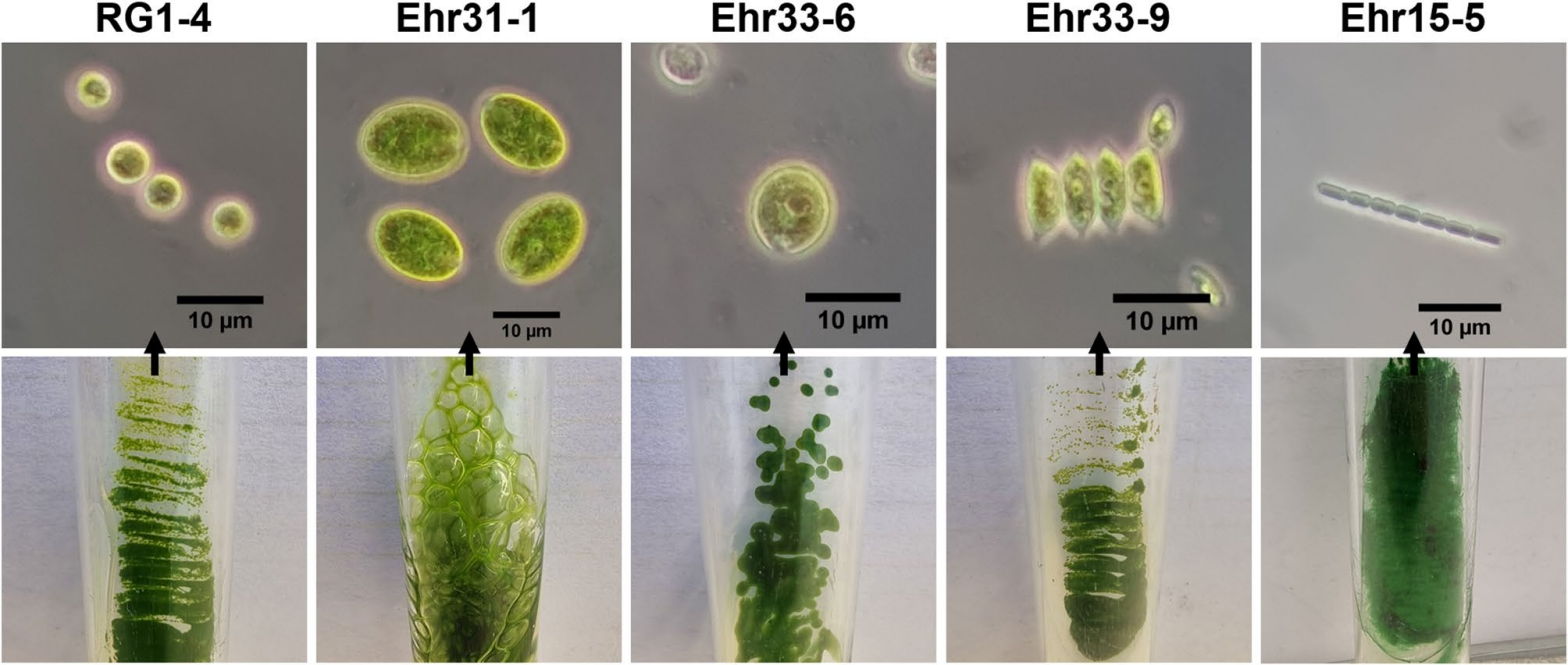
Phase contrast microscopic images (above) and agar medium culture pictures (below) of five selected isolates.

The strain Ehr31-1 was most resistant to Cd, while Ehr33-6 and Ehr33-9 showed the highest resistance to Cu. The isolate RG1-4 was notably resistant to both As(III), As(V), and Pb, with moderate tolerance to Cd, making it the most resilient green microalgae from the Roter Graben sampling location. Additionally, the cyanobacterial strain Ehr15-5 showed a high tolerance to Cr(VI), maintaining growth even exposed to 15 mg/L. These five bioprospected isolates were selected for further analysis. Their microscopic images are presented in Fig. 1.

### Morphological and molecular identification of selected isolates

Molecularly identified species and their morphological descriptions are given in Table 2.

**Table 2:** Identification and morphology of selected isolated species.

Isolate	Genes used for identification and their sequence GenBank accession no.	Identified species	Morphology
RG1-4	Combination of *rbcL* (PV805455), *tufA* gene (PV805456)	*Chlorella vulgaris* Beijerinck 1890	Cells are spherical, 2.9 to 6.4 μm in diameter, with cup-shaped chloroplasts that have one pyrenoid.
Ehr31-1	rRNA genes (PV823513)	*Lobochlamys segnis* (H. Ettl) Pröschold, B. Marin, U. W. Schlösser & Melkonian 2001	The cells are ellipsoid, measuring 10–12 μm by 6–8 μm. Each contains a large, rough-surfaced, cup-shaped chloroplast with a single pyrenoid near the base. The nucleus is slightly anterior. Asexual reproduction produces four zoospores, each of which can divide into two, resulting in up to eight zoospores.
Ehr33-6	ITS (PV823514)	*Chlamydomonas ulvaensis* R. A. Lewin 1957	This organism resembles *Chlamydomonas ulvaensis* morphologically, with a broad, ovoid cell size of 5.4 to 8.5 μm by 3.6 to 7.3 μm. The chloroplast does not extend to the flagella base, the nucleus is central, and the pyrenoid is slightly lateral. It lacks papillae and reproduces asexually, forming two to four zoospores.
Ehr33-9	Combination of *rbcL* (PV805459), *tufA* gene (PV805458)	*Tetradesmus obliquus* (Turpin) M. J. Wynne 2016	Cells are spindle-shaped and can be single, in pairs, or in groups of four, sometimes forming aggregates. Single cells measure 4.4 to 9.4 μm in length and 2 to 5.9 μm in width, while groups of four measure 8.1 to 14.5 μm by 5.4 to 10.0 μm. The chloroplast typically covers the entire cell and contains one pyrenoid, but in some single cells, it is located at one side.
Ehr15-5	16S rRNA (PV822044)	*Pseudanabaena catenata* Lauterborn 1915	Filamentous blue-green algae lack a sheath or mucilaginous layer. Active cultures show individual cells averaging 3.0 ± 0.7 μm in length and 1.5 ± 0.1 μm in width. The cylindrical cells typically have rounded ends and are longer than wide, but immediately after division, they are of similar length and width. Hyaline bridges connect the cells, and division occurs perpendicular to the filament’s long axis.

The isolates RG1-4 and Ehr33-9 successfully amplified the 18S rRNA, ITS, plastidial *rbcL* and *tufA* genes using the selected primer pairs. However, the highly conserved 18S rRNA gene does not resolve species-level differentiation, as seen with isolate Ehr33-9, which shares similarity with other *Scenedesmus* species. In contrast, the combination of the plastidial *rbcL* gene and *tufA* genes provides improved resolution for species differentiation, thus the use of the two genetic barcodes is recommended^31^.

#### Isolate RG1-4

Following the sequencing process, the lengths of the *rbcL* (GenBank accession no. PV805455) and *tufA* (PV805456) genes were determined to be 1,004 and 598 nucleotides (nt), respectively. These sequences were compared to other sequences in the GenBank in the NCBI database using the BLAST tool. The percentages of identity were found to be 99.80% and 100.00% (*Chlorella vulgaris* UTEX259 (MK948102.1)) for the *rbcL* and *tufA* genes, respectively. Then, the sequences from each result of the same strain were combined and aligned. The Maximum Likelihood (ML) tree was then inferred from the alignment with substitution model GTR+I+G. The isolate RG1-4 is more similar to *C. vulgaris* than other species and belongs to the same clade where other *C. vulgaris* strains belong to. Due to cell morphology, the percentage of identity and the ML tree inferred below, this isolate is further identified as *C. vulgaris* Beijerinck 1890 (Supplementary Figure S1).

#### Isolate Ehr31-1

The *rbcL* gene (PV805457) and SSU rRNA genes (PV823513) were sequenced for the Ehr31-1 isolate. However, few sequences with high similarity to the *rbcL* gene were found in GenBank, and the query coverage was low. Therefore, the small subunit ribosomal ribonucleic acid (SSU rRNA) genes (18S rRNA, ITS, partial 23S rRNA) were used for the phylogenetic analysis of this isolate (Supplementary Figure S2). The length of 2,488 nt sequence SSU genes were compared using BLAST. The SSU rRNA genes were found to be 98.96% similar to *Lobochlamys segnis strain* NH-29 (MT735192.1). In the ML tree, the isolate Ehr31-1 is in a clade which other branches of *Lobochlamys segnis*, (H. Ettl) Pröschold, B. Marin, U. W. Schlösser & Melkonian 2001 and is closely related to genus *Chlamydomonas*.

#### Isolate Ehr33-6

From this isolate, only the ITS sequence (PV823514) could be successfully amplified. According to NCBI BLAST result, the ITS sequence was most similar to *Chlamydomonas inflexa* CCAP 11/42 (FR865584.1) (99.16%) followed by *Chlamydomonas ulvaensis* SAG2.72 (AJ749612.1) (99.07%). However, these two sequences were 100% identical to each other. Only a few sequences of these two species were available in the GenBank database. The phylogenetic tree (see Supplementary Figure S3) shows that the Ehr33-6 isolate belongs to the same clade as *Chlamydomonas pulvinata* and the CCAP strains 11/166, 11/154 and 11/158. These isolates were 98.32–98.48% similar to Ehr33-6.

The genus *Chlamydomonas* is divided into nine species groups, which are defined by characteristics such as the shape of the chloroplast, the number of pyrenoids, and their positioning. *Chlamydomonas ulvaensis* belongs to the *Chlamydella *group, characterized by a cup-shaped chloroplast and one lateral pyrenoid. By contrast, *Chlamydomonas inflexa* belongs to the *Chlorogoniella* group, which has a chloroplast located on one side of the cell and one lateral pyrenoid^32^. The isolate Ehr33-6 exhibits a large, cup-shaped chloroplast and one lateral pyrenoid, indicating that it belongs to the *Chlamydella* group. Following the morphological characters in the identification key, it aligns with *Chlamydomonas ulvaensis* R. A. Lewin 1957.

#### Isolate Ehr33-9

After sequencing, the lengths of the *rbcL* (PV805459) and *tufA* (PV805458) genes were found to be 1,009 and 599 nucleotides, respectively. The best substitution model that was calculated by the JModeltest software was TIM2 + I + G (Akaike information criterion (AIC) was 19985.34). However, only a few nucleotide substitution models are available in the RAxML software. One of these models is GTR + I + G, which obtained the third best result (AIC was 19,987.41). Therefore, the GTR + I + G substitution model was used to infer the ML tree. The merged sequence was 99.5–100% similar to *Tetradesmus obliquus* strains. Moreover, the isolate Ehr33-9 belongs to the *Tetradesmus obliquus* clade in the inferred ML tree (Supplementary Figure S4). Consequently, Ehr33-9 can be categorised as *Tetradesmus obliquus* (Turpin) M.J. Wynne, 2016.

#### Isolate Ehr15-5

The partial 16S rRNA gene (PV822044) of isolate Ehr15-5 was polymerised and sequenced. This sequence was most similar to two different strains’, 99.64–99.93% similar to *Pseudanabaena catenata*’s 16S rRNA gene and 99.78–99.86% similar to *Pseudanabaena limnetica*’s 16S rRNA gene. In the ML tree, a *Synechococcus elongatus* PCC 630’s sequence was used as an outgroup. The best substitution model calculated by the JModeltest software was TIM1 + I + G (AIC was 7,232.75). However, the third best substitution model, GTR + I + G (AIC was 7,235.13) which was available in RAxML was used instead. The resulting ML tree is shown in Supplementary Figure S5.

Compared to the morphological descriptions of these two similar species in book^33^, Ehr15-5 is distinctly constricted between two adjacent cells in the filament (Fig. 1), resembling *Pseudanabaena catenata* more closely. Furthermore, the average cell width of Ehr15-5 is 1.5 ± 0.1 μm (with observed filaments reaching up to 1.8 μm wide), which is slightly wider than *Pseudanabaena limnetica*, which shows a width range of 1.2 to 1.5 μm. Based on these two differences and the molecular analyses, it was determined that the isolate Ehr15-5 is *Pseudanabaena catenata* Lauterborn 1915.

*T. obliquus*, *P. catenata*, and Chlamydomonas genus species were commonly found in Saxony. However, the “List of Algae Species in Saxony” from the State Office for Environment, Agriculture, and Geology did not include *L. segnis* or *C. ulvaensis*, and *C. vulgaris* had little evidence of presence between 1897 and 1989^34^. In the brown coal and lignite post-mining areas near Sokolov (Czech Republic) and Cottbus (Germany), *C. vulgaris* appeared in 25–50% of certain locations, alongside frequent occurrences of *Chlamydomonas* microalgae^35^. *C. vulgaris*, *T. obliquus*, and *C. reinhardtii* are well-studied organisms known for their rapid growth and ability to tolerate and uptake HMs^28,36^.

### HM tolerance of selected isolates

Microalgal growth was evaluated at HM concentrations of 0 to 10 mg/L, with cultures exposed up to 20 mg/L. Measurements were taken using optical density at 750 nm (OD_750_). As the metal concentrations increased, microalgal growth was inhibited compared to the control group (refer to Supplementary Figure S6). However, at lower metal concentrations, such as 0.5 mg/L of Cu and up to 2 mg/L of Cd for *C. vulgaris* RG1-4, growth was slightly enhanced compared to the control. *Asterarcys quadricellulare* and *Chlorococcum minutum* also showed increased growth at 0.4 mg/L of CdCl_2_, but growth declined at higher concentrations^37^. Among the tested microalgae, *C. vulgaris* RG1-4, *C. ulvaensis* Ehr33-6, and *T. obliquus* Ehr33-9 displayed the highest tolerance to copper, even at concentrations up to 20 mg/L. In contrast, *P. catenata* Ehr15-5, a cyanobacterium, died after 4 days at a concentration of 0.5 mg/L of copper. The microalga most tolerant to Cd exposure was *L. segnis* Ehr31-1; minimal adverse effects on growth were observed, even when Cd concentrations were elevated to 20 mg/L. However, Cr(VI) proved to be highly toxic to most microalgal species, with the cyanobacterium *P. catenata* Ehr15-5 showing the greatest tolerance.

**Fig. 2:**
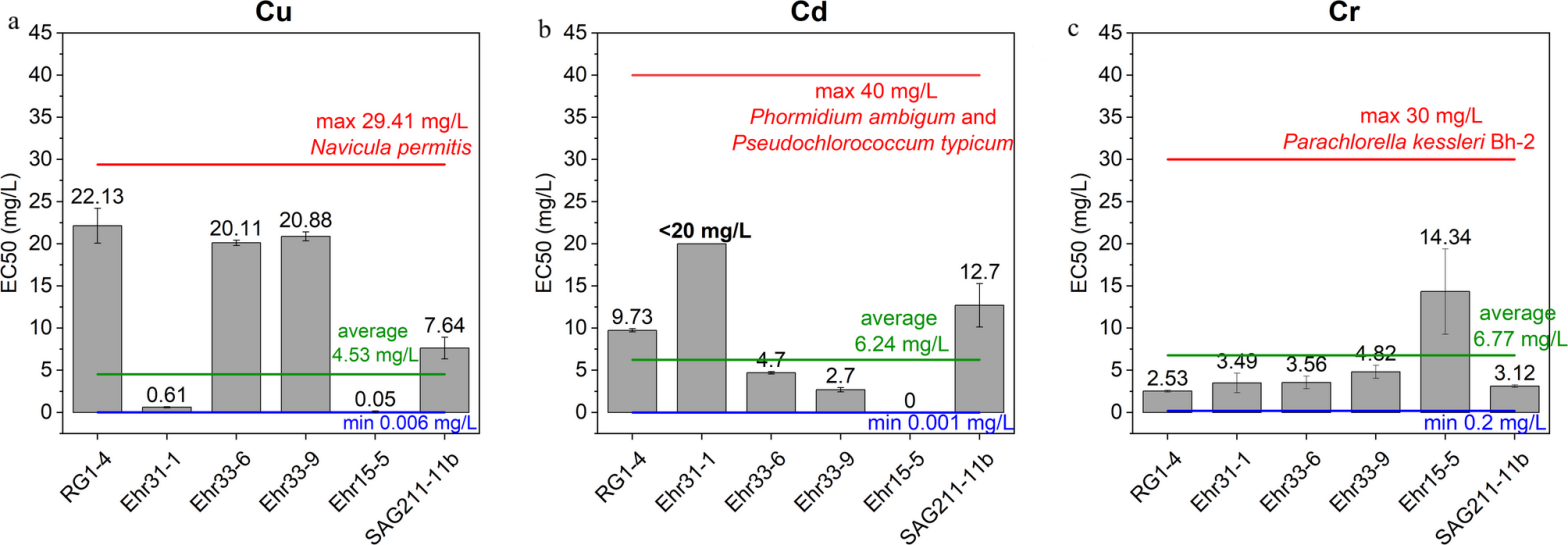
Comparison of EC50 values for metal tolerance among isolates: (**a**) Cu, (**b**) Cd, and (**c**) Cr(VI). The maximum, average, and minimum EC50 values found in literature are included. Here, SAG211-11b serves as the reference strain. The growth rate inhibition experiments were done in three biological replicates (*n* = 3) and the EC50 values were calculated by fitting the sigmoidal curve of Hill1 function.

From the growth curves, the half-maximal effective concentration (EC50) was determined (fitted curves are represented in the Supplementary Figure S7), and the findings were compared with values reported in previous studies (Fig. 2). Among the isolates tested for Cu tolerance, three exhibited the highest levels: *C. vulgaris* RG1-4, *C. ulvaensis* Ehr33-6, and *T. obliquus* Ehr33-9, with EC50 values of 22.13 ± 2.07 mg/L, 20.11 ± 0.32 mg/L, and 20.88 ± 0.52 mg/L, respectively. These values are significantly higher than the average EC50 value of 4.53 mg/L found in the literature (Supplementary Table S8). In contrast, the diatom isolate *Navicula permitis* demonstrated the highest EC50 value at 29.41 mg/L under similar conditions^38^. Notably, the same species of *C. vulgaris* and *T. obliquus* exhibited much lower tolerance to Cu, with EC50 values of only 0.1 mg/L^39^ and 5.84 mg/L^40^, respectively. In another study, *T. obliquus* showed an even lower EC50 value of 0.05 mg/L^41^. Other copper-tolerant microalgae included *Scenedesmus quadricauda* (CPCC 158), *Chlamydomonas reinhardtii* (CPCC 243), and *Dunaliella salina*, with corresponding EC50 values of 19.97 mg/L, 23.30 mg/L^42^, and 18.14 mg/L^43^, respectively.

Two isolates demonstrated higher tolerance to Cd than the average EC50 value of 6.24 mg/L reported in the literature (Fig. 2). The isolate *L. segnis* Ehr31-1 showed no growth inhibition at a Cd concentration of 20 mg/L, displaying only a 1.33% reduction in growth compared to the control after 96 h. This indicates that the isolate *L. segnis* Ehr31-1 can tolerate Cd concentrations exceeding 20 mg/L. The isolate *C. vulgaris* RG1-4 showed an EC50 value of 9.73 ± 0.19 mg/L, which is lower than the reference strain *C. vulgaris* SAG211-11b, which exhibited an EC50 of 12.70 ± 2.58 mg/L. One study reported that two strains of cyanobacteria could tolerate 40 mg/L of Cd^44^, but previously reported EC50 values were lower than the current results (Supplementary Table S9).

None of the isolated and reference green microalgae showed tolerance to high chromium concentrations, except for strains *C. ulvaensis* Ehr33-6 and *T. obliquus* Ehr33-9, which grew at 2 mg/L without significant negative effects. The cyanobacterium *P. catenata* Ehr15-5 displayed the highest tolerance, experiencing only minor effects at up to 10 mg/L, with an EC50 of 14.34 ± 5.06 mg/L. However, it could not tolerate Cu or Cd at 0.5 mg/L, leading to cell death and bleaching. Conversely, strain *C. vulgaris* RG1-4 was the least tolerant, with an EC50 of 2.53 ± 0.12 mg/L. Most literature reported lower EC50 values (Supplementary Table S10). However, *Craticula subminuscula* was able to tolerate Cr(VI) up to EC50 of 15.12 mg/L^45^ and *Phaeodactylum tricornutum* up to EC50 of 15.38 mg/L^46^; both species are diatoms. Furthermore, *Parachlorella kessleri* Bh-2 was identified in the literature as the most tolerant microalgae, with an EC50 of 30 mg/L^47^. Cr(VI) can be particularly toxic as it easily penetrates and disrupts cell membranes^48^.

Turning metals into less toxic forms is the one of the strategies of algae to alleviate HM toxicity. One possible reason why the cyanobacterium *P. catenata* Ehr15-5 can tolerate Cr(VI) is that it may reduced Cr(VI) to Cr(III) using its own chromium reductase, in a manner similar to that observed in *Spirulina* sp.^49^. Lee et al. found that live *C. vulgaris* was more effective at reducing Cr(VI) than dead cells, while its chromium reductase activity was lower than that of other bacteria^50^, and Cr(III) is less toxic than Cr(VI) as it is less soluble and more difficult to penetrate cell membranes^51^.

### HM removal of selected isolates

**Fig. 3:**
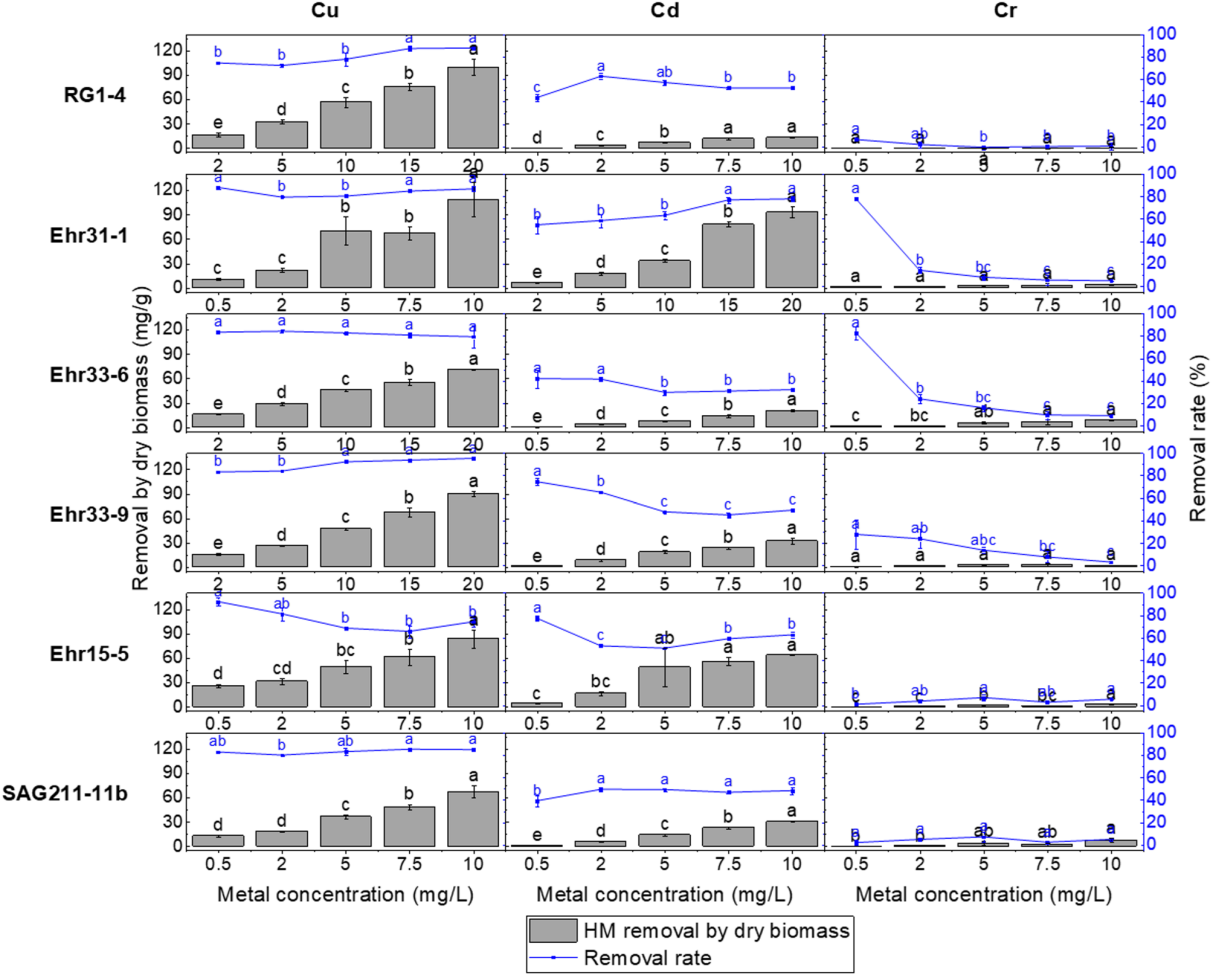
Heavy metal removal after 96 h of cultivation. The left axis shows the removal rate per dry biomass of microalgae (mg/g), while the right axis displays the percentage of heavy metal removal (%) relative to the initial concentration. The x-axis represents the heavy metal concentration. The experiments were done in three replicates (*n* = 3). Different letters above bars indicate statistically significant differences among HM concentrations (*p* < 0.5, one-way ANOVA with post-hoc Tukey’s test).

The extent of HM absorption by microalgal cells was measured in two ways: as a percentage of the total HM removed by the microalgal culture removal rate and as the amount of HM absorbed per dry biomass (see Fig. 3). The raw data from the ICP-MS measurements, including HM concentrations, dry biomass, and the calculated removal rates along with removal efficiency calculated per dry biomass, are presented in Supplementary Tables S1 to S6. The results of the overall one-way ANOVA test can be found in Supplementary Table S7. While the microalgae generally tolerated HMs well, their uptake typically increased with higher metal concentrations. Notably, the isolate *P. catenata* Ehr15-5 demonstrated good tolerance to hexavalent chromium but exhibited the lowest uptake of this metal. Similarly, the isolate *C. vulgaris* RG1-4 and the reference strain *C. vulgaris* SAG211-11b also showed a poor uptake performance (as indicated in the third column of Fig. 3).

The removal rate of Cu was highest in *T. obliquus* Ehr33-9 at 95.26 ± 0.46% and second highest in *C. vulgaris* RG1-4 at 87.92 ± 1.45%, both measured at a concentration of 20 mg/L of copper. The uptake per dry biomass for *T. obliquus* Ehr33-9 was 89.73 ± 2.77 mg/g, while *C. vulgaris* RG1-4 exhibited a higher uptake of 100.00 ± 9.52 mg/g. Their growth inhibition rates were 48.21 ± 3.39% and 40.05 ± 5.03%, respectively. These values were significantly higher than the uptakes reported for fresh biomass, which were 69.41 mg/g for *T. obliquus*^52^, 9.89 mg/g for *C. vulgaris*^53^, and 15.89 mg/g for -80°C stored *C. vulgaris*^54^. These experiments were conducted under optimal conditions, including appropriate pH, temperature, metal concentration, and algal dosage. When the copper concentration was 10 mg/L, the isolate *L. segnis* Ehr31-1 showed the highest absorption, with 108.85 ± 21.45 mg/g in terms of uptake per dry biomass. However, the growth of *L. segnis* Ehr31-1 was nearly completely inhibited (almost 100%) starting from a concentration of 2 mg/L of Cu (see Supplementary Figure S6). At low concentrations, Cu is an essential trace element for cells, playing crucial roles in photosynthesis, cellular respiration, the formation of superoxide dismutase with anti-ROS activity, and in the transport of iron across membranes^55^. However, at high concentrations, copper can damage the membranes of microalgae like *Chlorella sorokiniana* and *Scenedesmus acuminatus*, resulting in increased antioxidant activity^56^.

**Fig. 4:**
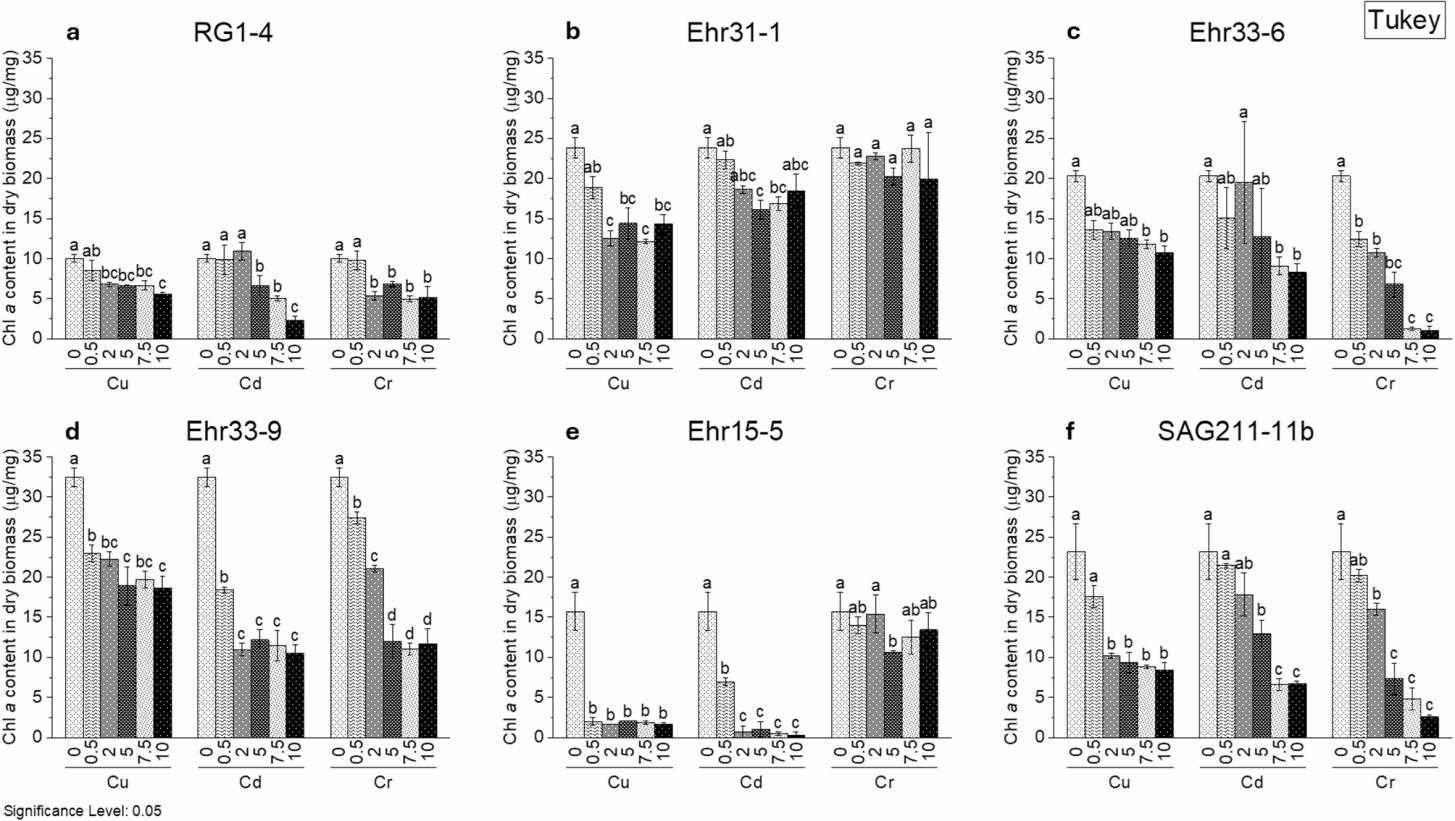
Chlorophyll *a* content in dry biomass treated with different concentration of heavy metals. Here, (**a**) RG1-4, (**b**) Ehr31-1, (**c**) Ehr33-6, (**d**) Ehr33-9, (**e**) Ehr15-5, (**f**) *C. vulgaris* SAG211-11b – reference strain. The heavy metals and their nominal concentrations are given on the x-axis. The experiments were done in three replicates (*n* = 3). Different letters above bars indicate statistically significant differences among HM concentrations (*p* < 0.5, one-way ANOVA with post-hoc Tukey’s test).

The Cd removal rate was highest for *L*. *segnis* Ehr31-1, achieving 77.86 ± 2.40% at a Cd concentration of 20 mg/L, with a cadmium removal per dry biomass of 93.17 ± 6.39 mg/g. Importantly, the growth of this isolate was inhibited by only 1.33% compared to the control. The experiment was carried out with Cd concentrations ranging up to 20 mg/L, but it seems that this isolate can tolerate higher concentrations and absorb more cadmium than the tested levels. When comparing cadmium removal per dry weight, *L*. *segnis* Ehr31-1 outperformed other similar species. For instance, the fresh biomass of *Chlamydomonas reinhardtii* showed a removal rate of 42.6 mg/g^57^, while the dried biomass of *Chlamydomonas* sp. recorded 44.75 mg/g^58^. *Chlamydomonas reinhardtii* immobilized in alginate beads demonstrated a higher rate of 79.7 mg/g^59^. Additionally, *P. catenata* Ehr15-5 was tested with 10 mg/L Cd, resulting in a removal rate of 62.56 ± 2.96% and a dry weight absorption of 63.51 ± 0.22 mg/g of cadmium. This microalga also showed a strong capacity for copper absorption, as illustrated in Fig. 3. However, its growth was significantly inhibited by 92.26 ± 5.80% at 0.5 mg/L copper and completely inhibited at 0.5 mg/L Cd (as shown in Supplementary Figure S6). Figure 4 demonstrated that its chlorophyll content was nearly absent, indicating negative impact on the cell physiology. For other species, *C. vulgaris* RG1-4 and *T. obliquus* Ehr33-9 removed 52.15 ± 1.12% and 49.31 ± 1.68% of Cd, respectively, with absorption per dry biomass of 13.32 ± 0.37 mg/g and 32.15 ± 3.35 mg/g. Their growth was inhibited by over 50% at 10 mg/L Cd. However, these absorption capacities were still lower than those of *C. vulgaris*, which had a freeze-stored biomass absorption of 15.89 mg/g^54^ and a dried biomass absorption of 85.3 mg/g^60^. *T. obliquus* had a freeze-dried biomass absorption of 68.6 mg/g^61^, while *D. pleiomorphus* (ACOI 561) also recorded a dried biomass absorption of 76.4 mg/g^62^. In a study by Nourbala Tafti et al., the addition of Fe_3_O_4_ nanoparticles to *C. vulgaris* and *T. obliquus* significantly enhanced their Cd uptake capacity, reaching 280.8 mg/g and 331.4 mg/g, respectively^63^.

The highest Cr(VI) removal was observed in *C. ulvaensis* Ehr33-6 and *L. segnis* Ehr31-1, with absorption rates of 82.65 ± 5.99% and 78.00 ± 1.52%, respectively, at a concentration of 0.5 mg/L. However, as the concentration of Cr(VI) increased, their absorption capacities decreased, and their specific growth rates were inhibited by about 30% at this concentration. At a Cr(VI) concentration of 10 mg/L, the absorption capacities were measured at 8.92 ± 0.32 mg/g for *C. ulvaensis* Ehr33-6 and 3.94 ± 0.18 mg/g for *L. segnis* Ehr31-1. These values are relatively low compared to other studies; for instance, dried *Chlamydomonas* sp. was reported to absorb 152 mg/g Cr(VI)^64^, dried *C. vulgaris* absorbed 14.78 mg/g, and *Scenedesmus acutus* absorbed 20.08 mg/g^65^. The fresh biomass of *Chlamydomonas reinhardtii* showed an absorption capacity of 18.2 mg/g, which increased to 25.6 mg/g after drying^66^. *P. catenata* Ehr15-5 showed some tolerance to Cr but absorbed just small amounts. Foster et al. noted that irradiation increased extracellular polysaccharide production^67^, hindering Cr(VI) uptake due to the negatively charged exopolysaccharides secreted by it^68^.

No microalgae were found to effectively absorb all three HMs. However, the highest uptake of HMs was observed in *C. vulgaris* RG1-4 and *T. obliquus* Ehr33-9 for Cu, with less growth inhibition. For Cd, *L. segnis* Ehr31-1 exhibited the best absorption performance. Therefore, these bioprospected isolates can potentially be used for bioremediation of Cu and Cd. Additionally, hexavalent Cr was best absorbed by *L. segnis* Ehr31-1 and *C. ulvaensis* Ehr33-6 at a concentration of 0.5 mg/L. While *P. catenata* Ehr15-5 showed the highest tolerance to Cr(VI), it absorbed Cr(VI) less effectively than the other isolates. It is evident that the tolerance and absorption of these HMs varied among different microalgae species.

Extracellular polymeric substances (EPS) are secreted by algal cells and consist of polysaccharides, proteins, DNA, humic substances, and inorganic ions. They surround algal cells and serve as a defensive mechanism^69^. Studies have shown that among the components of EPS isolated from sludge, Cu and Cd are more effectively adsorbed by proteins. The amide I and amide II groups of proteins, along with the carbonyl (C = O) and aromatic (C = C) stretching of humic acids, contribute to the sorption of these metals^70^. Shi et al. studied the changes in EPS from *Chlorella vulgaris* in response to Cu exposure. They found that the secretion of protein-like substances increased in loosely bound (soluble) EPS when exposed to Cu. Interestingly, at low Cu concentrations, the amount of polysaccharides in EPS increased. However, as Cu concentrations increased, the amount of polysaccharides decreased while the protein content in loosely bound EPS steadily increased. Under normal conditions, proteins are primarily located in tightly bound EPS^71^. Additionally, when three different groups of algae, green alga *Uronema confervicolum*, the cyanobacteria *Phormidium autumnale*, and the diatom *Nitzschia palea*—were exposed to Cu and Zn, the protein content in their EPS increased at high concentrations of these metals^72^. This can be one of the possible explanations of why *C. vulgaris* RG1-4 and *T. obliquus* Ehr33-9 exhibited greater tolerance to high concentrations of Cu.

*L. segnis* Ehr31-1, in comparison to RG1-4 and Ehr33-9, formed mucoid colonies on agar medium (Fig. 1). It also generated a slimy biomass that adhered to the inner walls of conical flasks of old cultures in liquid medium. This phenomenon may occur because the oxygen produced by photosynthesis cannot escape, leading to the formation of bubbles on solid medium due to the secretion of strong polysaccharides. The primary component of the outer capsule or biomass of *L. segnis* SAG1.79 is chlamyhyaluronic acid, and its production increases under stress conditions^73^. In the second column of Fig. 1, four closely located cells can be seen within a single capsule. Recently, Visconte et al. studied *L. segnis* SAG1.79 and found that under stress conditions, a capsular polysaccharide (CPS) similar to hyaluronan was produced in large quantities. This CPS exhibited a highly branched random coil structure and demonstrated reducing power, antioxidant activity, and thickening properties, with the capability of secreting up to 1 g/L of EPS under light stress conditions^74^. Hyaluronan is typically produced by vertebrates and a few pathogenic bacteria; it is also produced in the *Chlorella* genus of microalgae when infected by phycodnaviruses^75^. Due to these differences, the capsule of *L. segnis* Ehr31-1 may have absorbed a significant amount of Cd to protect the cells from Cd toxicity.

### Chlorophyll *a* content in dry biomass

When measuring growth through optical density, some cultures exhibited a visual decrease in green colour, yet their optical density values suggested good growth. Chl *a* is a crucial photosynthetic pigment found in all organisms that perform oxygenic photosynthesis. Besides measuring optical density, Chl *a* can serve as an additional indicator of HM impacts on cell physiology. Figure 4 demonstrates the correlation between varying concentrations of HMs and the alteration of Chl *a* levels within the dry biomass. Chl *a* concentration in dry weight was measured at the end of cultivation, specifically for treatments with Cu, Cd, and Cr(VI) at a concentration of 10 mg/L. Despite some microalgal cultures showing higher optical density than controls at low HM concentrations (see Supplementary Figure S6), Chl *a* content in dry biomass was consistently lower than controls when exposed to HMs. Notably, isolates Ehr33-6 and Ehr33-9 significantly reduced Chl *a* content even at low Cu and Cr(VI) levels. While Cd had minimal impact on strain Ehr31-1 growth, Chl *a* content slightly decreased as Cd concentration increased.

The changes in Chl *a* content observed in *C. vulgaris* RG1-4, *P. catenata* Ehr15-5, and *C. vulgaris* SAG211-11b were consistent with the changes noted in optical density. However, in *L. segnis* Ehr31-1, Cr(VI) reduced the optical density without significantly impacting the Chl *a* content in the dry biomass. This suggests that while the cells did not grow well, they remained intact.

In another study involving *Scenedesmus quadricauda*, it was found that Chl *a* content decreased as the concentration of Cr(VI) increased from 5 ppm to 10 ppm^76^. Additionally, increases in Cu and Cd concentrations above 0.014 mg/L and 0.04 mg/L, respectively, also decreased the Chl content^77^. For the microalgae *Asterarcys quadricellulare* and *Chlorococcum minutum*, treated with Cd at a concentration of 0.4 mg/L, both cell growth and total chlorophyll content increased compared to the control (TAP medium). However, as Cd concentration rose, both cell growth and total chlorophyll content decreased^37^. This trend was also observed in *C. reinhardtii*, *C. luteoviridis*, and *P. kessleri*^78^, as well as in various aquatic plants^79^ and bryophytes^80^. Nevertheless, a study by Ye et al. demonstrated that when *C. reinhardtii* was exposed to half the EC50 concentration of Cu for 96 h, the chlorophyll content was higher than that of the control group^81^.

The decrease in Chl *a* content may be linked to various cellular changes induced by HMs. These changes may include elevated levels of ROS, metal accumulation in chloroplasts, disruption of membrane ion balance, damage to thylakoid membranes, reduced photosynthetic activity, and alterations in gene expression resulting from these damages^36,82^. For instance, in *C. reinhardtii*, exposure to Cd for a short period led to a decrease in the expression of the enzyme ferredoxin NADP^+^ reductase, which was associated with a reduction in chlorophyll content. Furthermore, prolonged exposure to Cd significantly reduced the expression of enzymes involved in the synthesis of chlorophyll and carotenoids^83^. Similarly, in the brown macroalga *Ectocarpus siliculosus*, short-term copper treatment resulted in the repression of enzymes such as chlorophyll synthase, Mg chelatase, and protoporphyrinogen oxidase, all of which play roles in the steps of chlorophyll synthesis^84^. In these studies, protective protein expression was also up-regulated. The decrease in chlorophyll may be attributed to the allocation of more cellular resources and energy toward protective activities, resulting in fewer resources and energy available for other normal functions.

### Future research directions

The isolates *C. vulgaris* RG1-4 and *T. obliquus* Ehr33-9 were able to absorb higher amounts of copper (Cu), while *L. segnis* Ehr31-1 demonstrated a greater capacity to absorb cadmium (Cd). Therefore, these isolates can be utilized for treating wastewater containing Cu and Cd as the primary pollutants. The main sources of Cd pollution include nickel-cadmium (Ni-Cd) batteries and the electroplating industries^85^. In contrast, higher concentrations of Cu are typically found in wastewater from copper mining^86^.

To further enhance the HM biosorption capacity of these isolates, several studies could be conducted. Potential approaches might include chemically treating the biomass, supplementing the growth medium to boost the production of EPS, providing additional sulfur and phosphate to strengthen the cells’ biosorption, and genetically engineering the cells to increase metallothionein production, enhance EPS production, or even display metal-binding proteins on their surfaces^87–89^. Kalita and Baruah found that treating *Leptolyngbya* sp. GUEco 1015 with 0.1 mM CaCl_2_ and 0.3 mM HCl reinforced the microalgae’s filament surface, enabling better Cu absorption^90^. Similarly, adding more sulfate to the medium of *Chlamydomonas moewusii* resulted in increased production thiol compounds, thereby enhancing Cd tolerance^91^. By supplementing medium with additional phosphorus, the microalgae can produce more polyphosphate bodies, leading to increased sequestration of Cd and Zn in species like *Chlamydomonas reinhardtii*^92^ and *Tetradesmus obliquus*^93^. Additionally, genetically engineering *Chlamydomonas reinhardtii* to display metalloregulatory protein (MerR) allowed it to uptake five times more Hg than the wild type^94^.

When developing these isolates for field applications, three key considerations must be taken into account. First, it is essential to understand the kinetics and isotherm behavior of metal adsorption by these isolates. Adsorption typically occurs rapidly within the first 20 to 30 min of contact with metals, after which the rate slows until equilibrium is reached. This information can help determine the optimal duration for using these isolates to treat metal contaminants. Understanding the isotherm will provide insights into the maximum adsorption capacity and the binding characteristics of the isolates^52,53,95^. Second, it is crucial to test these isolates under various conditions, including changes in pH, temperature, salinity, and the presence of co-existing metal ions, to evaluate their effects on metal binding rates and capacities^88,96^. Third, if these isolates are used in the field, they will need to be harvested. Given that these isolates are single-celled organisms with a diameter ranging from 4 μm to 12 μm, harvesting can be challenging and costly. To overcome this obstacle, methods such as immobilization^97^ using alginate^98^, water-hyacinth derived pellets^99^, or loofah sponge^100^ can be employed. Additionally, microalgae can be magnetized by using Fe_3_O_4_ nanoparticles, enabling easy harvesting with magnets^63^.

## Conclusion

Since the increase in industrialization worldwide, heavy metals have been released into the environment due to human activities. Their presence poses a significant threat to living organisms and can lead to biomagnification in the food chain, which in turn presents a high risk to human health. One environmentally friendly and cost-effective method to reduce heavy metal pollution is the use of microalgae. In a search for suitable microalgae in old mining areas of Saxony, Germany, Cu-absorbing strains of *Chlorella vulgaris* RG1-4 and *Tetradesmus obliquus* Ehr33-9, along with a Cd-absorbing strain of *Lobochlamys segnis* Ehr31-1, were successfully isolated. These strains demonstrate a high capacity to absorb Cu and Cd compared to other strains, making them promising candidates for treating wastewater primarily contaminated with these heavy metals. Future research should focus on understanding the kinetics and isotherms of metal uptake, as well as how factors such as pH, temperature, and contact time influence their ability to absorb metals. Additionally, exploring the possibility of immobilizing these microalgae for practical applications could enhance their effectiveness in pollution mitigation.

## Methods

### Sampling locations

**Fig. 5:**
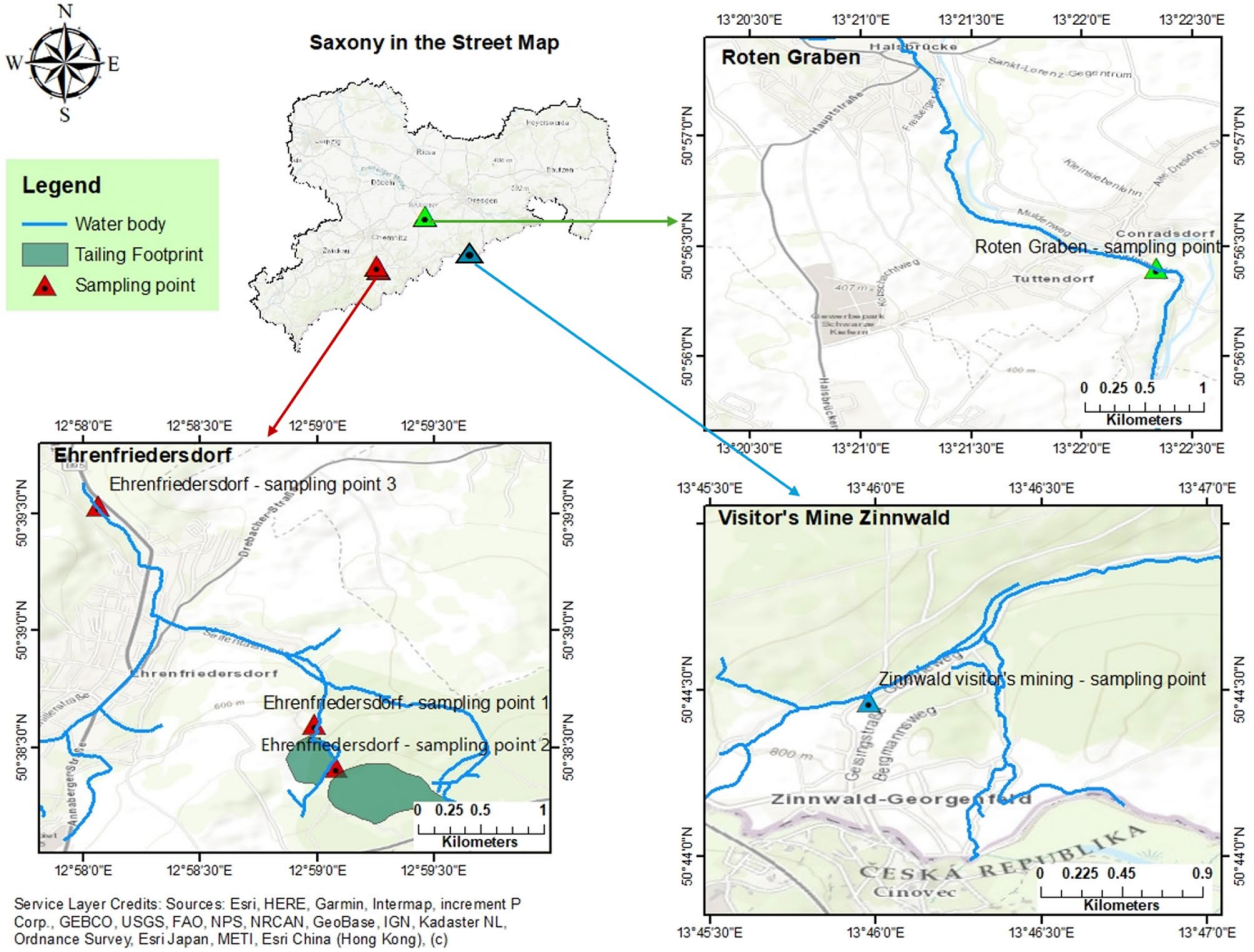
Sampling locations in Erzgebirge, Saxony. The basemap utilized is the World Street Map, and the credits for the basemap are indicated at the bottom left^102^. The water body data was taken from the River Basin Districts and Catchment Boundaries in Germany by Esri Deutschland (© Bundesamt für Naturschutz (BfN) 2023)^103^.

Samples were taken from three locations, Roter Graben near Freiberg (50°56’24.1"N 13°22’20.1"E), three other locations near Ehrenfriedersdorf (around 50°38’36.0"N 12°58’59.6"E) and Visitor’s Mine Zinnwald (50°74’11.6"N,13°76’35.9"E) from Erzgebirge (Ore Mountains) in south-eastern Germany (Saxony). Sampling locations are shown on the map (Fig. 5). The map was created using ArcGIS Desktop version 10.8 software (Environmental Systems Research Institute, Inc. (Esri))^101^. Sample collection was conducted in accordance with local regulations, and no special permission was required.

The Erzgebirge has several metals that are mined from the 12th to the 20th century. The silver ore was discovered first, and it was mined continuously from 1168 to 1968. Between 1460 and 1560, the Erzgebirge was the most renowned silver producer in Europe. The second metal that was extracted from the region was a tin. Then the region became an important uranium producer in the world at the end of 19th century. During the 800 years of mining, the landscape of Erzgebirge had shaped a lot. Therefore, the Erzgebirge region was registered as a UNESCO World Heritage Site in 2019^29,101^.

### Isolation and selection of HM tolerant microalgae

#### Isolation of pure microalgal culture

To enrich the microalgae from the samples, they were cultured in both solid media containing 1.5% agar and liquid media: BG11, Bold’s Basal Medium (BBM), Spirulina medium^102^, and Z8 medium^103–105^. Microalgal colonies were collected with a fine needle and streak-plated onto new solid media for isolation. However, repeated streak-plating was often insufficient to eliminate bacteria from most of the isolates. Consequently, the microalgae were treated with various antibiotics.

Single antibiotics were insufficient to eliminate various types of bacteria and fungi, so multiple antibiotics were tested for their effectiveness without harming microalgal growth. These included Penicillin (25–1000 µg/mL), Streptomycin (10–250 µg/mL), Gentamicin (10–250 µg/mL), Kanamycin (10–250 µg/mL), Chloramphenicol (5–100 µg/mL), and Cycloheximide (5–100 µg/mL). After selecting up to three antibiotics at the appropriate concentration, they were applied sequentially for 24 h each.

The antibiotics mentioned above function by inhibiting cell wall formation and protein synthesis and they work well on actively growing cells. Especially, antibiotics that inhibit the cell wall formation kill bacteria that are only actively dividing. Therefore a small amount of nutrients should be added to enhance bacterial growth^102^. An sterile nutrient solution (SNS) was added (2 mL of SNS solution into 100 mL medium), consisting of 2.5% sucrose, 0.5% Difco yeast extract, and 0.5% Difco Bacto-Peptone^106^. After each 24-hour treatment, most of the bacteria were reduced, but not eliminated. Thus, from each treatment, 20 µL samples were streaked onto suitable solid media. The colonies were then grown in liquid media, and their purity was examined by culturing them in BII, BIV, and BV media containing organic sources^107^. The presence of bacteria was assessed under a microscope (Axioskop-2 MOT, Carl Zeiss, Oberkochen, Germany) to confirm their absence.

### Selection of microalgal cultures that are tolerant to high concentrations of HMs

The isolated strains’ and as references *Chlorella vulgaris* SAG 211-11b, and *Limnospira maxima* CCALA 27’s tolerance to high concentration of heavy HM(oid)s: As(III) – 100 mg/L (NaAsO_2_), As(V) – 100 mg/L (Na_2_HAsO_4_), Cd – 10 mg/L (Cd(NO_3_)_2_ · 4H_2_O), Cr(VI) – 15 mg/L (K_2_Cr_2_O_7_), Cu – 8 mg/L (CuSO_4_ · 5H_2_O), and Pb – 20 mg/L (Pb(NO_3_)_2_) were determined. The concentrations used were based on research articles assessing microalgal tolerance to HMs^108^ and the culture media BBM and Z8 were prepared without EDTA due to its chelating property of metals^109,110^. The experiment was conducted in a 24-well plate with a working volume of 1 mL (Nunc™ cell culture multi-dishes, treated, Thermo Fisher Scientific, Waltham, MA, USA). Microalgal growth was measured as OD_750_ (optical density at 750 nm) daily by a plate reader (Infinite^®^ 200 PRO, TECAN, Männedorf, Switzerland)^111^. Microalgae were grown for 4 days in the same culture cabinet. The experiment was done in 3 replicates. For green microalgae, the average of 3 wells’ optical density which containing normal medium, and microalgae after 4 days was considered as 100% of growth. The growth rates of other wells were then compared to this percentage. Cyanobacteria were observed for growth in a medium containing HM, comparing it to the control, with observations made visually.

### Morphological and molecular identification

#### Morphological identification

The morphology of selected isolates was observed under Axioskop-2 MOT microscope. The morphological features of each isolates were compared to identification keys^32,33,112^. Morphological identification in higher levels of taxonomy helped to choose the right primer pairs for further molecular identification.

#### Genomic DNA isolation

Genomic DNA from the cultures was isolated using Chelex-100^113,114^. When cells are treated with Chelex-100 at 100°C, they are disrupted, releasing the DNA into the media. Chelex-100 acts as a chelating agent, binding magnesium ions, which are cofactors for deoxyribonucleases. This binding protects the released DNA from degradation. From the fresh liquid culture, 3 mL samples were taken and centrifuged using refrigerated centrifuge (Sigma 3-30KHS, Sigma Laborzentrifugen GmbH, Osterode am Harz, Germany) at 14,000×g. The supernatant was discarded, and 200 µL of a 5% Chelex-100 solution (pH 8.0, in 1X TE buffer) was added to the pellet in a 1.5 mL Eppendorf tube. Because Chelex-100 tends to precipitate easily, the solution was thoroughly mixed using a magnetic stirrer while pipetting. The mixture of fresh biomass and Chelex-100 was then vortexed and heated in a water bath at 100°C for 15 min with an additional vortex at the 8-minute mark. Following this, the tubes were centrifuged at 12,000×g for 90 s at room temperature. The supernatant was transferred to a new tube, avoiding any Chelex resin. The DNA concentration and purity were measured using a Infinite^®^ 200 PRO microplate reader. Finally, the extracted DNA was stored at -20°C.

#### Polymerase chain reaction (PCR) and sequencing

For molecular identification SSU genes (18S rRNA, ITS, partial 23S rRNA), ITS gene, and plastidial *rbcL* and *tufA* genes were used for identification of green microalgal isolates and 16S rRNA gene used for cyanobacterial isolate. The primer pairs used for PCR (polymerase chain reaction) are given in Table 3.

**Table 3:** Detailed information about primers.

Target gene	Primer	Sequence	Product length, nt	Reference
*rbcL*	RcbLZ-F	5’-CAACCAGGTGTTCCASCTGAAG-3’	≈ 1323	^31^
	RcbLZ-R	5’-CTAAAGCTGGCATGTGCCATAC-3’		
*tufA*	tufA-SF	5’-TGGATGGTGCWATTYTWG-3’	789	^31^
	tufA-SR	5’-GGTTTTGCWAAAACCATWCCACG-3’		
ITS	ITS-AF	5’-CGTTTCCGTAGGTGAACCTGC-3’	≈ 700	^115^
	ITS-BR	5’-CATATGCTTAAGTTCAGCGGGT-3’		
SSU (18 S rRNA, ITS)	EAF3 – Fw	5’-TCGACAATCTGGTTGATCCTGCCAG-3’	≈ 2400	^116^
	ITS055R - Rv	5’-CTCCTTGGTCCGTGTTTCAAGACGGG-3’		
16 S rRNA	27 F - fw	5’-AGAGTTTGATCCTGGCTCAG-3’	≈ 1480	^117^
	16 C - rv	5’-AAGGAGGTGATCCAGCCGCA-3’		^118^

PCR (Polymerase Chain Reaction) mix: 5x Phusion HF or GC buffer 4 µL, 10 mM dNTPs 0.4 µL, 10 µM forward primer 1 µL, 10 µM reverse primer 1 µL, Phusion DNA polymerase 0.2 µL (New England Biolabs GmbH), template DNA 1 µL, ddH2O until the volume reaches 20 µL. The PCR was conducted in thermocycler (peqSTAR 96X, PEQLAB Biotechnologie GmbH, Erlangen, Germany) and the PCR condition was: Initial denaturation at 98°C for 30s, 35 cycles of denaturation at 98°C for 10s, annealing at appropriate temperature calculated by Tm Calculator v.1.16.6^119^ for each primer pairs, extension at 72°C for 1 min and final extension at 72°C for 10 min. The PCR products were kept at -20°C.

PCR products were cleaned by Monarch^®^ PCR & DNA Cleanup Kit T1030 (New England Biolabs GmbH) – to obtain only PCR produced DNA fragments. The DNA fragments’ sequences were sequenced by Sanger sequencing method in GENEWIZ (Azenta Life Sciences, Leipzig, Germany). And sequences were submitted to GenBank, then the Accession numbers are given in the results.

#### Data analysis and phylogenetic tree

Sequences were manually edited using the BioEdit v7.2 software^120^. Then, NCBI BLASTn (Basic Local Alignment Search Tool) tool^121^ was used to align isolates’ sequences with other sequences in GenBank database. Then some of the sequences from this alignment were used for further phylogenetic analysis. The alignment is done by Clustal Omega software^122^. Model of nucleotides substitution selected by AIC method by jModelTest v2.1.10 software^123^. The Maximum Likelihood tree inferred on this alignment and nucleotide substitution model by RAxML v8.2 software^124^ and illustrated by FigTree v1.4.4 software^125^.

### Toxicity test of selected isolates in different concentrations of HM

#### The experimental setup

The selected microalgae were grown in medium with different concentrations of HM, Cu (0.5–10 mg/L or 2–20 mg/L) (CuSO_4_·5H_2_O), Cd (0.5–10 mg/L or 2–20 mg/L) (Cd(NO_3_)_2_·4H_2_O), Cr(VI) (0.5–10 mg/L) (K_2_Cr_2_O_7_). For simplicity, the text refers only to the concentration of the HM (e.g., Cd instead of CdCl_2_) and not to the weighed salt. The growth was monitored for 96 h, during which the growth conditions were maintained at 25°C with shaking at 150 rpm. The light/dark cycle was 14/10 hours with a light intensity ranging from 46 to 65 µmol/(m^2^s). A total of 50 mL of culture was prepared in a 100 mL conical flask using a chelator (EDTA) - free medium: BBM for green microalgae and Z8 medium for cyanobacteria. In the HM tolerance test, microalgae growth was measured daily at OD_750_. In addition, chlorophyll *a* content, dry biomass weight, and pH levels were assessed at both the beginning and the end of the test. Furthermore, 10 mL of filtered samples were collected at the experimental endpoint for subsequent elemental analysis and stored at -20 °C. The experiments were done in three biological replicates.

#### Inoculum preparation

Inoculum cultures were prepared by growing green microalgae in a bubble column and a cyanobacterium in a CellDEG system (CellDEG GmbH, Berlin, Germany). The bubble column utilized mixed red and blue LED light at intensities of 50 to 100 µmol/(m^2^s), starting lower and gradually increasing as the culture turned green. Mixing was achieved with a magnetic stirrer at 150 rpm, and aeration was provided via a sparger with 0.45 μm filtered air. Cultures were incubated in a sterile environment for 5 to 6 days. Cyanobacteria in the CellDEG system were illuminated with white LED light at 20 to 30 µmol/(m^2^s). They were maintained in 150 mL vessels at 125 rpm, aerated with 3% CO₂ for 7 days. Cultures in the exponential growth phase were then used for HM tolerance experiments.

Inoculum preparation for the HM resistance assay commenced with an initial concentration of OD_750_ = 0.1. The inoculum was separated from the culture medium by centrifugation at 5000 rpm for 10 min using a centrifuge (Megafuge 16R, Thermo Fisher Scientific, Osterode am Harz, Germany) and washed twice with sterile distilled water (dH_2_O).

#### Growth measurement

Each day, a 1 mL sample was collected and thoroughly mixed with a pipette if necessary. The sample was then analysed for OD_750_ using a UV-VIS spectrophotometer (GENESYS™ 180, Thermo Fisher Scientific, Waltham, MA, USA). Blanks were prepared using the medium without a chelator, as well as the same medium with each concentrations of HMs. A growth curve was generated from these measurements, and growth inhibition (GI) was calculated using the following equations^126^:

1 $$\:{K}_{HM}=\frac{ln{OD}_{t2}-ln{OD}_{t1}}{{t}_{2}-{t}_{1}}$$

2 $$\:{K}_{C}=\frac{ln{OD}_{Ct2}-ln{OD}_{Ct1}}{{t}_{2}-{t}_{1}}$$

3 $$\:GI=\frac{{K}_{HM}}{{K}_{C}}\times\:100\%\:$$

Where:

*OD*_*t1*_ – optical density at day 0.

*OD*_*t2*_ – optical density at day 4.

*t*_*2*_ *– t*_*1*_ – cultivation period (4 days).

*K*_*HM*_ – specific growth rate when microalgae treated with HM.

*K*_*C*_ – specific growth rate when microalgae grown in the control.

*GI* – growth inhibition.

The growth inhibition for each concentration of HM is plotted and the EC50 value was calculated by fitting sigmoidal curve by Hill1 function on OriginPro 2025 software^127^. This function’s sigmoidal curve equation is given below.

4 $$\:y=a+\frac{b-a}{1+{\left(\frac{k}{x}\right)}^{n}}$$

Where:

*a* – minimum asymptote/growth inhibition when HM concentration is 0 mg/L, indicating that the algae were cultured in the control medium. Therefore, this value has been manually set to 0%.

*b* – maximum asymptote/growth inhibition when HM concentration is high, indicating complete growth suppression. Therefore, this value has been manually set to 100%.

*k* – the HM concentration at which 50% of the microalgal population are expected to show growth inhibition (e.g. EC50 value).

*n* – the slope at the steepest part of the curve (Hill slope).

#### Chlorophyll *a* measurement

Chl *a* content was measured at the start and end of the experiment and expressed as Chl *a* content in dry biomass. It was measured when isolates where treated with HMs up to 10 mg/L concentration. Pigment extraction followed the method in reference^128^. A 1 mL of the sample was transferred to a 2 mL tube and centrifuged at 14,000 g for 10 min using a Megafuge 16R centrifuge. After discarding the supernatant, the pellet was resuspended in 1 mL DMSO, vortexed, and incubated at 70°C and 700 rpm for 10 min in a Thermomixer (Thermomixer Comfort 5355, Eppendorf SE, Hamburg, Germany). Following incubation, the mixture was centrifuged again at 14,000 g for 10 min, and the supernatant was transferred to a new 2 mL tube. The pellet underwent the same treatment with DMSO, and the resulting supernatant was combined with the previous one. Then the absorption of this 2 × diluted solution measured at wavelengths of 649 nm, 665 nm (664 nm for cyanobacteria), and 750 nm using a spectrophotometer. The Chl *a* content of green microalgae was calculated using Eq. 5^129^, while the Chl *a* content of cyanobacteria was calculated using Eq. 6^130^:

5 $$\:{C}_{a}\left(\mu\:g\:{mL}^{-1}\right)=12.19{A}_{665nm}-3.45{A}_{649nm}$$

6 $$\:{C}_{a}\left(\mu\:g\:{mL}^{-1}\right)=11.4063\times\:{A}_{664\:}$$

#### Dry weight measurement

Dry weight was measured for inoculum and at the end of the experiment. A 20 mL of the culture was filtered using filtered using glass fibre filters (ROTILABO^®^, Type CR261, Carl Roth GmbH + Co. KG, Karlsruhe, Germany) with a vacuum pump. After drying at 60°C for 24 h, the filtered material was cooled in a desiccator for 40 min, and then the dry weight was measured.

#### HM removal measurement

At the end of the experiment (after 96 h), the 10 mL of the remaining filtered solutions were acidified with 500 µL concentrated HNO_3_ (~ 65%) and stored at -20 °C for further analysis of HM concentrations. Samples’ metal concentration were analysed by an inductively coupled plasma mass spectrometer (iCAP™ RQ ICP-MS, Thermo Fisher Scientific Inc., Waltham, MA, USA) at Institute of Resource Ecology, Helmholtz Zentrum Dresden Rossendorf.

The details of ICP-MS calibration procedures were: Scandium (Sc) and rhodium (Rh) were used as internal standards. The linearity of the calibration curve (R^2^ values) was 1.000 for all elements. The limits of detection were 0.01 µg/L, 0.001 µg/L, and 0.068 µg/L for Cu, Cd, and Cr, respectively. The quantification limits were 0.25 µg/L, 0.1 µg/L, and 0.1 µg/L for Cu, Cd, and Cr, respectively. The data on recovery or reproducibility were 1.28%, 1.40%, and 1.42% for Cu, Cd, and Cr, respectively.

HM removal was expressed in two ways, removal per dry biomass (mg/g) and removal rate (%). The HM concentrations for the calculation were the ICP-MS measured concentrations. And they were calculated accordingly:

7 $$\:RB(mg/g)=\frac{{C}_{HM}\left(before\right){-C}_{HM}\left(after\right)}{DW}$$

8 $$\:RR\left(\%\right)=\frac{{C}_{HM}\left(before\right){-C}_{HM}\left(after\right)}{{C}_{HM}\left(before\right)}\times\:100\%$$

Where:

*RB(mg/g)* – removal per dry biomass.

*C*_*HM*_*(before)* – HM concentration at the beginning of the treatment, mg/L.

*C*_*HM*_*(after)* – HM concentration at the end of the treatment, mg/L.

*DW* – dry weight of microalgal biomass per 1 L culture.

*RR(%)* – removal rate.

### Statistical analysis

Experiments were conducted with three biological replicates (*n* = 3). Statistical analysis was conducted using the statistical package of OriginPro 2025 software^127^. One-way ANOVA with Tukey’s post hoc test was used to evaluate treatment significance at a 5% level (p-value). Data are presented as mean ± standard deviation (mean ± S.D.).

### Writing, editing, and proofreading

Grammarly and ChatGPT were used only to produce error-free English text with clear and coherent composition. All analyses, interpretations, and scientific content were developed and verified by the authors.

## Supplementary Information

Below is the link to the electronic supplementary material.

Supplementary Material 1

## Data Availability

All sequences obtained for the identification of algal isolates have been deposited in a public sequence repository [GenBank – INSDC] under the following accession numbers: *Chlorella vulgaris* RG1-4 *rbcL* (PV805455.1), *tufA* (PV805456.1); *Lobochlamys segnis* Ehr31-1 rRNA genes (PV823513.1); *Chlamydomonas ulvaensis* Ehr33-6 ITS (PV823514.1); *Tetradesmus obliquus* Ehr33-9 *rbcL* (PV805459.1), *tufA* (PV805458.1); *Pseudanabaena catenata* Ehr15-5 16S rRNA (PV822044.1). Data will be made available on request.
