# Supplementary material for: Isolation and characterization of heavy metal tolerant microalgae from old mining areas of Saxony
Source: Sci Rep. 2026 Jan 5;16:1337. doi: 10.1038/s41598-025-32393-0 (PMC12796387; doi:10.1038/s41598-025-32393-0)
Supplement: Supplementary file 1 — Supplementary Material 1 [file 41598_2025_32393_MOESM1_ESM.pdf]

Isolation and characterization of heavy metal tolerant microalgae from old mining areas of Saxony

Khongorzul Mungunkhuyag[1,2], Juliane Steingroewer[1], Thomas Walther[1], and Felix Krujatz[3,*]

[1]Chair of Bioprocess Engineering, Institute of Natural Materials Technology, Technische Universität Dresden, 01069, Germany

[2]Department of Biology, School of Arts and Sciences, National University of Mongolia, Ulan-Bator, 14200, Mongolia

[3]biotopa gGmbH, Radeberg, 01454, Germany

[4] Professorship Automatic Control and System Dynamics, Technische Universität Chemnitz, Reichenhainer Straße 70, 09126 Chemnitz, Germany

*corresponding author: felix.krujatz@etit.tu-chemnitz.de

Supplementary Figure S1.

Maximum Likelihood tree of isolate RG1-4 based on the combination of *rbcL* and *tufA* genes. The best model of substitution was GTR+I+G (base frequencies: A 0.2978, C 0.1704, G 0.2173, T 0.3146; rate matrix A-C 0.6313, A-G 2.0642, A-T 2.7491, C-G 0.7162, C-T 6.1307, G-T 1.0000) with proportion of invariation p-inv = 0.2550 and gamma shape parameter 0.4640. Here, *Chlamydomonas reinhardtii* CC-373 was used as an outgroup.

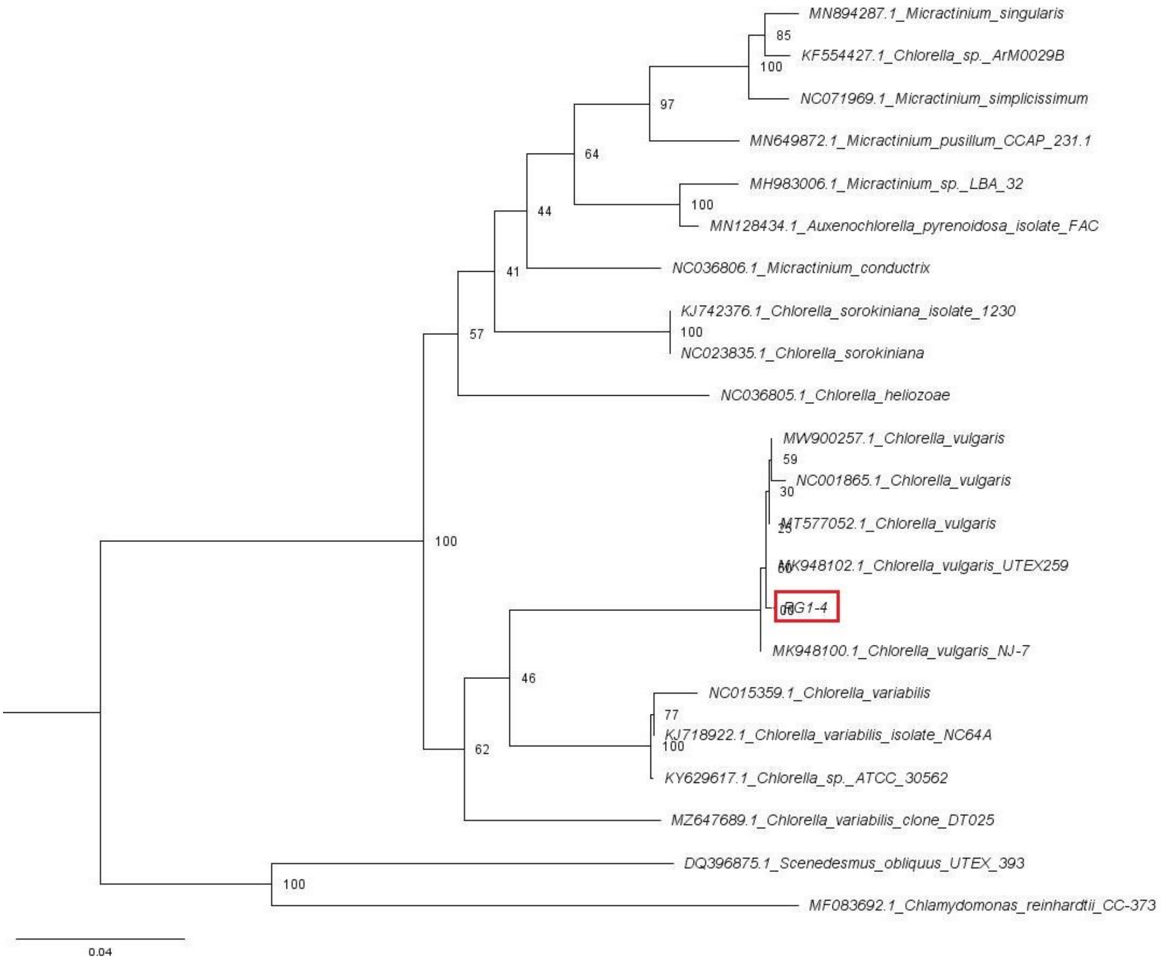

Supplementary Figure S2.

Maximum Likelihood tree of isolate Ehr31-1 based on the combination of rRNA genes. The best model of substitution was GTR+I+G (base frequencies: A 0.2384, C 0.2365, G 0.2637, T 0.2614; rate matrix A-C 1.4450, A-G 2.2576, A-T 1.7926, C-G 0.6855, C-T 4.0580, G-T 1.0000) with proportion of invariation p-inv = 0.4630 and gamma shape parameter 0.3530. Here, *Chlorella vulgaris SAG 211-11b* and *Tetradesmus obliquus* CCAP 276/1A were used as outgroup.

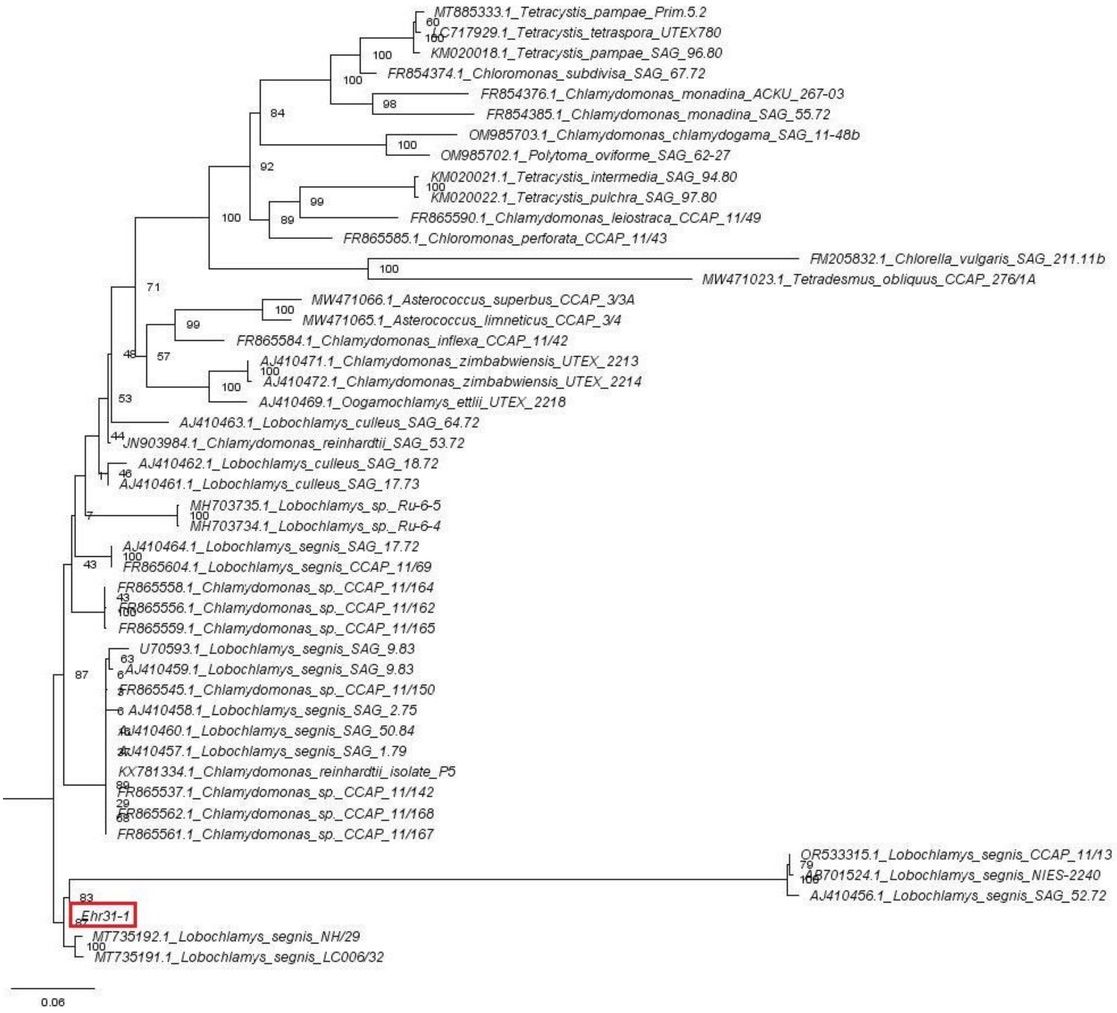

Supplementary Figure S3.

Maximum Likelihood tree of isolate Ehr33-6 based on ITS region sequence. The best model of substitution was GTR+G (base frequencies: A 0.2203, C 0.2878, G 0.2356, T 0.2564; rate matrix A-C 1.8455, A-G 2.5315, A-T 2.3911, C-G 0.3960, C-T 3.9321, G-T 1.0000) with gamma shape parameter 0.3030. Here, *Chlorella vulgaris* CCAP211/51 was used as an outgroup.

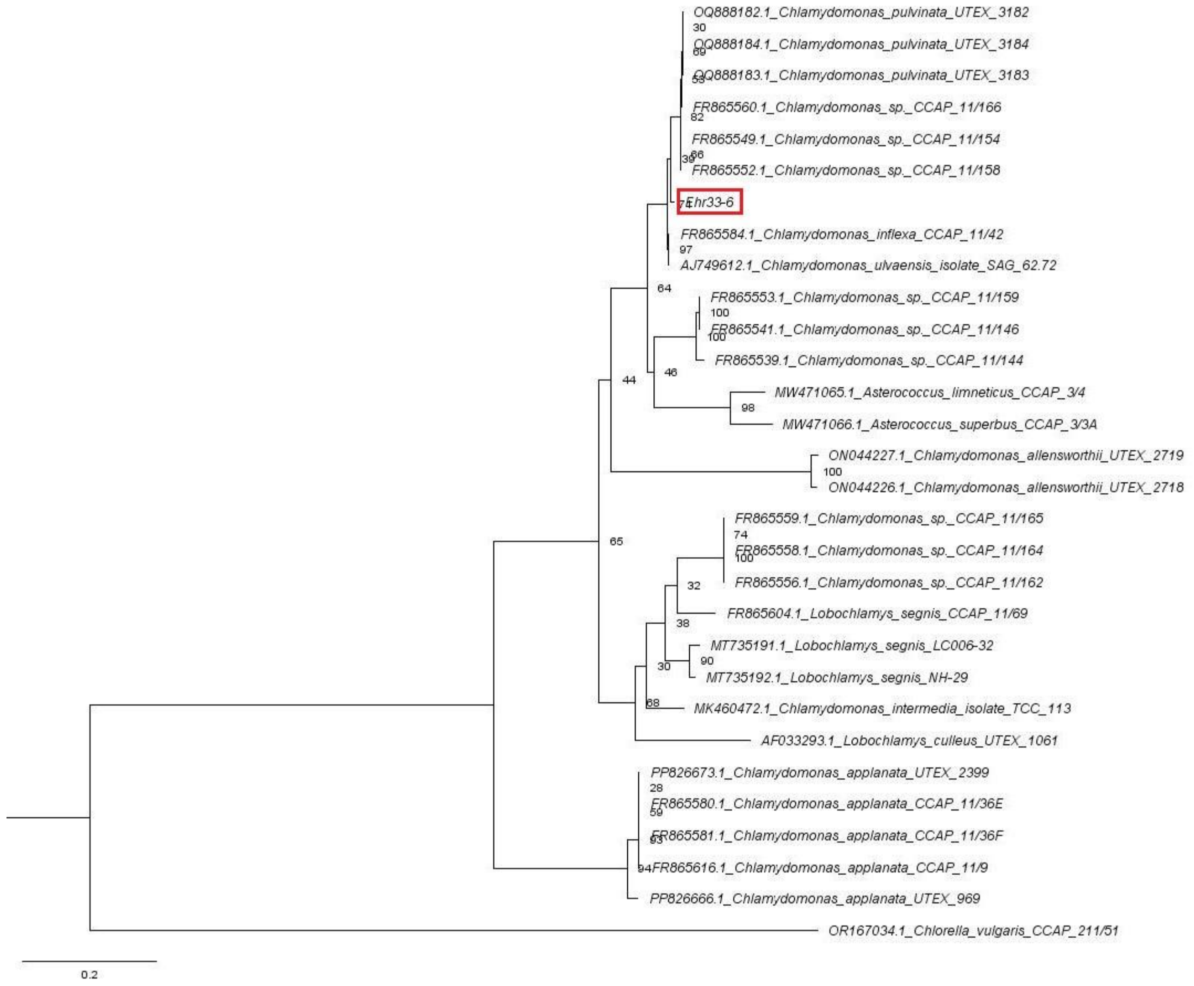

Supplementary Figure S4

Maximum Likelihood tree of isolate Ehr33-9 based on merged *rbcL* and *tufA* gene sequence. The model of substitution was GTR+I+G (base frequencies: A 0.2461, C 0.2246, G 0.2732, T 0.2562; rate matrix A-C 1.4103, A-G 2.9223, A-T 1.4103, C-G 1.0000, C-T 6.0698, G-T 1.0000) with proportion of invariation p-inv = 0.5540 and gamma shape parameter 0.4230. Here, *Chlorella vulgaris* UTEX259 and *Chlamydomonas reinhardtii* CC-373 were used as an outgroup.

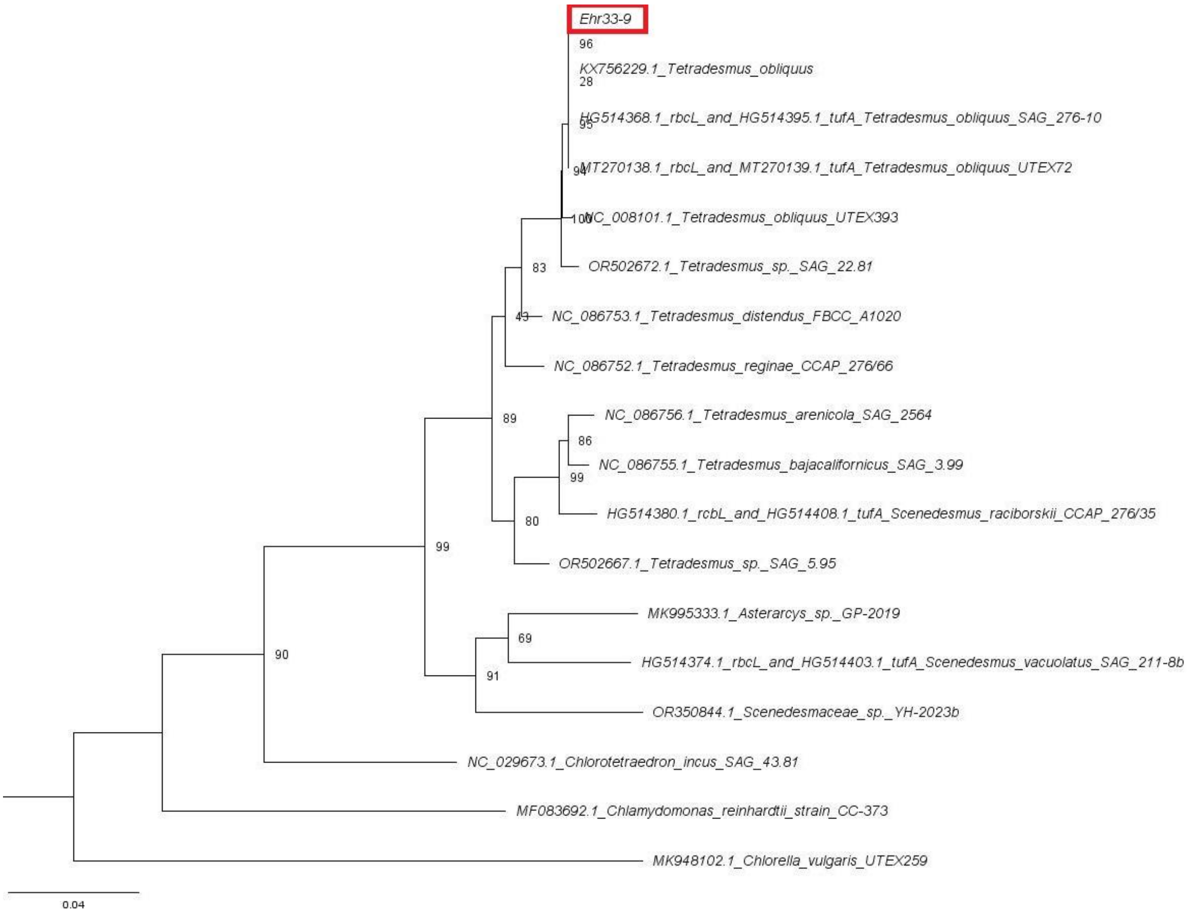

Supplementary Figure S5

Maximum Likelihood tree of isolate Ehr15-5 based on 16S rRNA gene sequence. The model of substitution was GTR+I+G (base frequencies: A 0.2572, C 0.2215, G 0.3110, T 0.2104; rate matrix A-C 1.0000, A-G 3.1169, A-T 1.8159, C-G 1.8159, C-T 8.9408, G-T 1.0000) with proportion of invariation p-inv = 0.6910 and gamma shape parameter 0.3570. Here, *Synechococcus elongatus* PCC6301 was used as an outgroup.

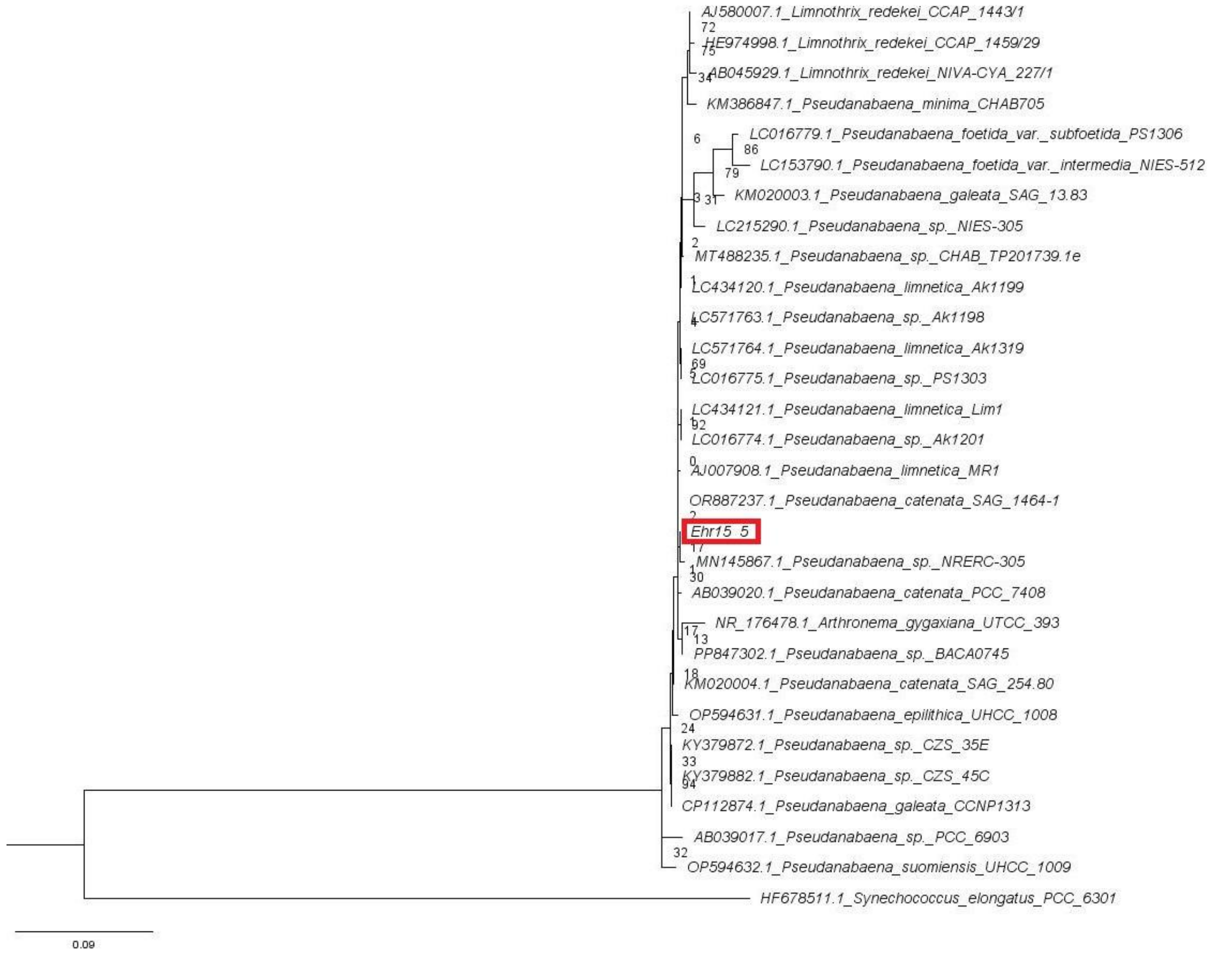

Supplementary Figure S6

Growth curve of isolates treated with heavy metals; Cu, Cd, and Cr. Algae growth was measured by optical density at 750 nm wavelength, OD_{750}. The heavy metal treatment continued for 96 h. In the legend above each graph the heavy metal concentrations are given in mg/L. Most of the treatment were tested until 10 mg/L of heavy metal and some were tested until 20 mg/L of heavy metal (Cu for RG1-4, Ehr31-1, Ehr33-9 and Cd for Ehr31-1). Each treatment was performed with three biological replicates (n=3), and error bars represent standard deviation.

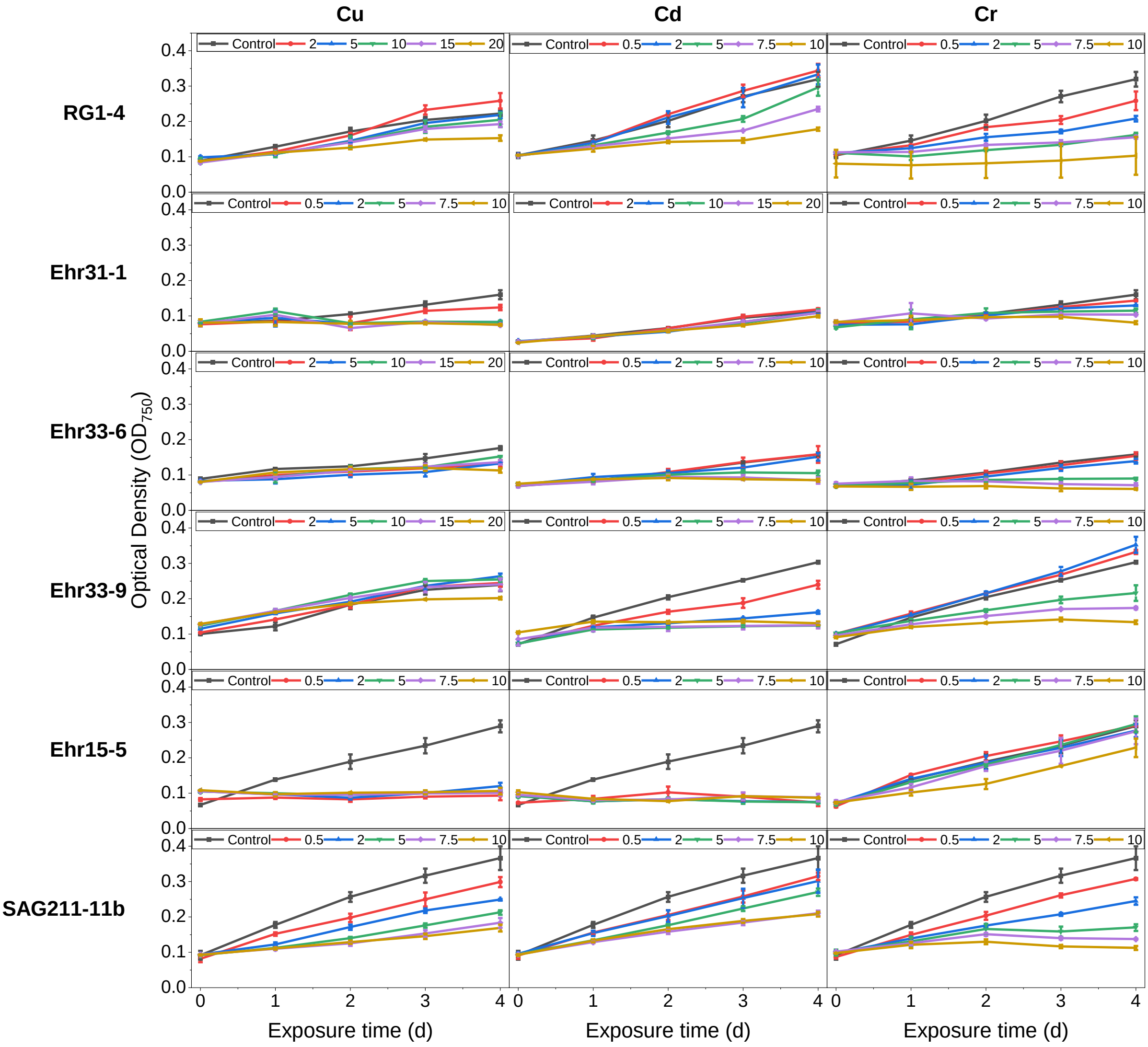

Supplementary Figure S7.

Growth inhibition of algal isolates exposed to increasing concentrations of Cd, fitted with a sigmoidal Hill1 model to determine EC50 values. For each curve, the fitted EC50 value (k), the Hill slope (n), Reduced Chi-square value, coefficient of determination (R-Square (COD)), and how well a model fits the data (Adj. R-Square) are shown. It was not possible to fit sigmoidal curve for some of the inhibition data (e) and n)), because in e) until 20 mg/L of Cd there was no significant reduction in the relative growth rate and in n) the algal growth was completely inhibited in the lowest concentration, 0.5 mg/L, of Cd. Reliable sigmoidal fits were obtained for RG1-4 (a – c), Ehr31-1 (d – f), Ehr33-6 (g – i), Ehr33-9 (j – l), Ehr15-5 (m – o), and SAG211-11b (p – r). Sigmoidal fitting was not possible for panels (e) and (n): in (e), no significant inhibition was observed up to the highest tested concentration (20 mg/L), resulting in an undetermined EC50 (EC50 > 20 mg/L); in (n), growth was completely inhibited at the lowest tested concentration (0.5 mg/L), giving EC50 < 0.5 mg/L. Each treatment was performed with three biological replicates (n = 3), and error bars represent standard deviation.

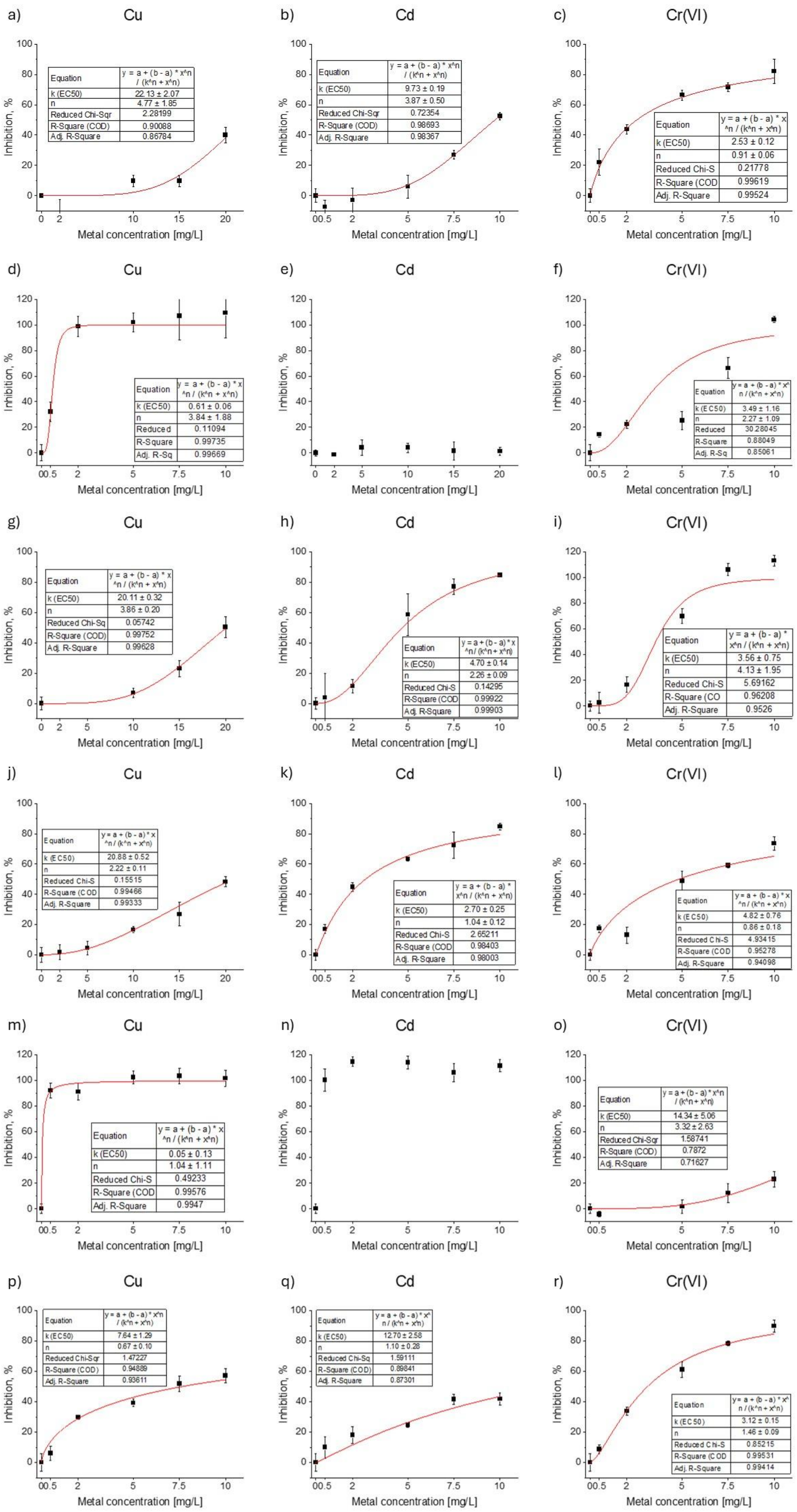

a) Cu
Equation y = a + (b - a) * x^n / (k^n + x^n)
k (EC50) 22.13 ± 2.07
n 4.77 ± 1.85
Reduced Chi-Sqr 2.28199
R-Square (COD) 0.90088
Adj. R-Square 0.86784
b) Cd
Equation y = a + (b - a) * x^n / (k^n + x^n)
k (EC50) 9.73 ± 0.19
n 3.87 ± 0.50
Reduced Chi-Sqr 0.72354
R-Square (COD) 0.98693
Adj. R-Square 0.98367
c) Cr(VI)
Equation y = a + (b - a) * x ^n / (k^n + x^n)
k (EC50) 2.53 ± 0.12
n 0.91 ± 0.06
Reduced Chi-S 0.21778
R-Square (COD 0.99619
Adj. R-Square 0.99524
d) Cu
Equation y = a + (b - a) * x ^n / (k^n + x^n)
k (EC50) 0.61 ± 0.06
n 3.84 ± 1.88
Reduced 0.11094
R-Square 0.99735
Adj. R-Sq 0.99669
e) Cd
f) Cr(VI)
Equation y = a + (b - a) * x^ n / (k^n + x^n)
k (EC50) 3.49 ± 1.16
n 2.27 ± 1.09
Reduced 30.28045
R-Square 0.88049
Adj. R-Sq 0.85061
g) Cu
Equation y = a + (b - a) * x ^n / (k^n + x^n)
k (EC50) 20.11 ± 0.32
n 3.86 ± 0.20
Reduced Chi-Sq 0.05742
R-Square (COD) 0.99752
Adj. R-Square 0.99628
h) Cd
Equation y = a + (b - a) * x ^n / (k^n + x^n)
k (EC50) 4.70 ± 0.14
n 2.26 ± 0.09
Reduced Chi-S 0.14295
R-Square (COD 0.99922
Adj. R-Square 0.99903
i) Cr(VI)
Equation y = a + (b - a) * x^n / (k^n + x^n)
k (EC50) 3.56 ± 0.75
n 4.13 ± 1.95
Reduced Chi-S 5.69162
R-Square (CO 0.96208
Adj. R-Square 0.9526
j) Cu
Equation y = a + (b - a) * x ^n / (k^n + x^n)
k (EC50) 20.88 ± 0.52
n 2.22 ± 0.11
Reduced Chi-S 0.15515
R-Square (COD 0.99466
Adj. R-Square 0.99333
k) Cd
Equation y = a + (b - a) * x^n / (k^n + x^n)
k (EC50) 2.70 ± 0.25
n 1.04 ± 0.12
Reduced Chi-S 2.65211
R-Square (COD 0.98403
Adj. R-Square 0.98003
l) Cr(VI)
Equation y = a + (b - a) * x ^n / (k^n + x^n)
k (EC50) 4.82 ± 0.76
n 0.86 ± 0.18
Reduced Chi-S 4.93415
R-Square (COD 0.95278
Adj. R-Square 0.94098
m) Cu
Equation y = a + (b - a) * x ^n / (k^n + x^n)
k (EC50) 0.05 ± 0.13
n 1.04 ± 1.11
Reduced Chi-S 0.49233
R-Square (COD 0.99576
Adj. R-Square 0.9947
n) Cd
o) Cr(VI)
Equation y = a + (b - a) * x^n / (k^n + x^n)
k (EC50) 14.34 ± 5.06
n 3.32 ± 2.63
Reduced Chi-Sqr 1.58741
R-Square (COD) 0.7872
Adj. R-Square 0.71627
p) Cu
Equation y = a + (b - a) * x^n / (k^n + x^n)
k (EC50) 7.64 ± 1.29
n 0.67 ± 0.10
Reduced Chi-Sqr 1.47227
R-Square (COD) 0.94889
Adj. R-Square 0.93611
q) Cd
Equation y = a + (b - a) * x^ n / (k^n + x^n)
k (EC50) 12.70 ± 2.58
n 1.10 ± 0.28
Reduced Chi-Sq 1.59111
R-Square (COD) 0.89841
Adj. R-Square 0.87301
r) Cr(VI)
Equation y = a + (b - a) * x^ n / (k^n + x^n)
k (EC50) 3.12 ± 0.15
n 1.46 ± 0.09
Reduced Chi-S 0.85215
R-Square (COD 0.99531
Adj. R-Square 0.99414
Inhibition, %
Metal concentration [mg/L]

Supplementary Table S1

Raw data for calculating metal removal efficiency (%) and metal uptake capacity (mg/g dry weight) of *Chlorella vulgaris* RG1-4 is presented below. The data includes three replicates of metal concentrations measured by ICP-MS, along with the dry weight of the microalgae recorded at the end of the experiment (after 96 hours) by drying the filtrates in an oven at 60°C for 24 hours. In this study, the concentration of copper (Cu) was higher in the initial media concentrations due to the presence of Cu in the BBM (Bold's Basal Medium). Because Cu is an essential element for photosynthesis, it could not be removed from the media. The Cu concentration in the control medium was measured at 1.66 ± 0.01 mg/L using ICP-MS.

Metal		Initial concentration mg/L					Final metal concentration, mg/L					Removal efficiency, %					Dry weight, mg/L			Uptake capacity, mg/g				
	Nominal	replicates			average	std	replicates			average	std	replicates			average	std	replicates			replicates			Average	std
		1	2	3			1	2	3			1	2	3			1	2	3	1	2	3		
Cu	2	3.555	3.340	3.310	**3.402**	**0.134**	0.860	0.840	0.895	**0.865**	**0.028**	74.718	75.306	73.689	**74.571**	**0.818**	0.190	0.150	0.135	13.377	17.078	18.568	**16.341**	**2.673**
	5	5.900	5.950	5.900	**5.917**	**0.029**	1.640	1.550	1.720	**1.637**	**0.085**	72.282	73.803	70.930	**72.338**	**1.437**	0.125	0.130	0.140	34.213	33.590	29.976	**32.593**	**2.288**
	10	10.350	11.100	10.500	**10.650**	**0.397**	2.990	2.255	1.820	**2.355**	**0.591**	71.925	78.826	82.911	**77.887**	**5.553**	0.150	0.150	0.140	51.067	55.967	63.071	**56.702**	**6.036**
	15	13.900	14.000	13.800	**13.900**	**0.100**	1.800	2.040	1.445	**1.762**	**0.299**	87.050	85.324	89.604	**87.326**	**2.154**	0.150	0.165	0.165	80.667	71.879	75.485	**76.010**	**4.417**
	20	18.000	17.750	18.000	**17.917**	**0.144**	1.980	2.460	2.050	**2.163**	**0.259**	88.949	86.270	88.558	**87.926**	**1.447**	0.145	0.170	0.160	109.908	90.922	99.167	**99.999**	**9.521**
Cd	0.5	0.391	0.409	0.383	**0.394**	**0.013**	0.226	0.232	0.208	**0.222**	**0.012**	42.615	41.219	47.186	**43.673**	**3.121**	0.370	0.395	0.310	0.453	0.411	0.599	**0.488**	**0.099**
	2	1.570	1.615	1.585	**1.590**	**0.023**	0.635	0.570	0.570	**0.592**	**0.038**	60.063	64.151	64.151	**62.788**	**2.360**	0.275	0.279	0.289	3.468	3.657	3.534	**3.553**	**0.096**
	5	3.915	3.720	3.845	**3.827**	**0.099**	1.540	1.740	1.645	**1.642**	**0.100**	59.756	54.530	57.012	**57.099**	**2.614**	0.297	0.309	0.307	7.701	6.762	7.096	**7.186**	**0.476**
	7.5	5.650	5.500	5.800	**5.650**	**0.150**	2.760	2.675	2.675	**2.703**	**0.049**	51.150	52.655	52.655	**52.153**	**0.869**	0.285	0.253	0.221	10.131	11.775	13.446	**11.784**	**1.657**
	10	7.050	7.100	7.500	**7.217**	**0.247**	3.360	3.505	3.495	**3.453**	**0.081**	53.441	51.432	51.570	**52.148**	**1.122**	0.296	0.270	0.283	13.050	13.741	13.162	**13.318**	**0.371**
Cr	0.5	0.418	0.418	0.374	**0.403**	**0.026**	0.386	0.387	0.389	**0.387**	**0.002**	6.646	6.404	5.798	**6.283**	**0.437**	0.300	0.296	0.225	0.092	0.089	0.106	**0.096**	**0.009**
	2	1.685	1.660	1.685	**1.677**	**0.014**	1.605	1.680	1.665	**1.650**	**0.040**	4.496	0.033	0.926	**1.818**	**2.361**	0.316	0.281	0.274	0.239	0.002	0.057	**0.099**	**0.124**
	5	4.180	4.220	4.175	**4.192**	**0.025**	4.745	4.635	4.595	**4.658**	**0.078**	-2.496	-0.120	0.744	**-0.624**	**1.678**	0.206	0.193	0.229	-0.562	-0.029	0.151	**-0.147**	**0.371**
	7.5	6.450	6.500	6.300	**6.417**	**0.104**	6.750	6.700	6.850	**6.767**	**0.076**	0.328	1.066	-1.148	**0.082**	**1.128**	0.301	0.157	0.194	0.074	0.459	-0.401	**0.044**	**0.431**
	10	8.350	8.450	8.450	**8.417**	**0.058**	9.100	8.950	9.550	**9.200**	**0.312**	1.444	3.069	-3.430	**0.361**	**3.382**	0.212	0.186	0.135	0.630	1.522	-2.341	**-0.063**	**2.023**

Supplementary Table S2

Raw data for calculating metal removal efficiency (%) and metal uptake capacity (mg/g dry weight) of *Lobochlamys segnis* Ehr31-1 is presented below. The data includes three replicates of metal concentrations measured by ICP-MS, along with the dry weight of the microalgae recorded at the end of the experiment (after 96 hours) by drying the filtrates in an oven at 60°C for 24 hours. In this study, the concentration of copper (Cu) was higher in the initial media concentrations due to the presence of Cu in the BBM (Bold's Basal Medium). Because Cu is an essential element for photosynthesis, it could not be removed from the media. The Cu concentration in the control medium was measured at 1.66 ± 0.01 mg/L using ICP-MS.

Metal	Nominal	Initial concentration mg/L					Final metal concentration, mg/L					Removal efficiency, %					Dry weight, mg/L			Uptake capacity, mg/g				
		replicates 1	replicates 2	replicates 3	average	std	replicates 1	replicates 2	replicates 3	average	std	replicates 1	replicates 2	replicates 3	average	std	replicates 1	replicates 2	replicates 3	replicates 1	replicates 2	replicates 3	Average	std
Cu	0.5	2.150	2.165	2.055	**2.123**	**0.060**	0.253	0.232	0.280	**0.255**	**0.024**	88.085	89.097	86.837	88.006	1.132	0.187	0.162	0.202	9.988	11.654	9.113	10.252	1.291
	2	3.555	3.340	3.310	**3.402**	**0.134**	0.695	0.690	0.725	**0.703**	**0.019**	79.569	79.716	78.687	79.324	0.556	0.142	0.122	0.112	19.014	22.169	23.813	21.665	2.439
	5	5.900	5.950	5.900	**5.917**	**0.029**	1.145	1.160	1.185	**1.163**	**0.020**	80.648	80.394	79.972	80.338	0.342	0.127	0.082	0.057	37.49*	57.699	82.550	**70.124**	**17.572**
	7.5	8.250	8.250	7.550	**8.017**	**0.404**	1.220	1.220	1.245	**1.228**	**0.014**	84.782	84.782	84.470	84.678	0.180	0.117	0.092	0.097	57.959	73.690	69.656	67.102	8.171
	10	10.350	11.100	10.500	**10.650**	**0.397**	1.220	1.290	1.720	**1.410**	**0.271**	88.545	87.887	83.850	86.761	2.542	0.092	0.102	0.067	102.241	91.479	132.882	108.947	21.449
**Cd**	2	1.570	1.615	1.585	**1.590**	**0.023**	0.226	0.232	0.208	**0.222**	**0.012**	50.314	50.629	63.208	54.717	7.355	0.135	0.135	0.130	5.926	5.963	7.731	6.540	1.032
	5	3.915	3.720	3.845	**3.827**	**0.099**	0.635	0.570	0.570	**0.592**	**0.038**	64.983	57.012	53.354	58.449	5.946	0.130	0.125	0.125	19.128	17.453	16.333	17.638	1.407
	10	7.050	7.100	7.500	**7.217**	**0.247**	1.540	1.740	1.645	**1.642**	**0.100**	60.439	67.436	61.270	63.048	3.823	0.140	0.140	0.130	31.155	34.762	34.013	33.310	1.904
	15	12.950	13.700	12.950	**13.200**	**0.433**	2.760	2.675	2.675	**2.703**	**0.049**	79.091	74.015	77.614	76.907	2.611	0.135	0.120	0.135	77.333	81.417	75.889	78.213	2.867
	20	17.550	17.350	17.600	**17.500**	**0.132**	3.360	3.505	3.495	**3.453**	**0.081**	75.686	80.429	77.457	77.857	2.397	0.150	0.155	0.135	88.300	90.806	104.407	93.171	6.391
Cr	0.5	0.418	0.418	0.374	**0.403**	**0.026**	0.090	0.085	0.098	**0.091**	**0.006**	78.205	79.416	76.389	78.003	1.524	0.192	0.177	0.192	1.679	1.848	1.640	1.722	0.111
	2	1.685	1.660	1.685	**1.677**	**0.014**	1.390	1.465	1.470	**1.442**	**0.045**	17.289	12.826	12.529	14.215	2.667	0.172	0.147	0.162	1.685	1.462	1.297	1.481	0.195
	5	4.180	4.220	4.175	**4.192**	**0.025**	4.370	4.285	4.125	**4.260**	**0.124**	5.604	7.440	10.896	7.980	2.687	0.167	0.157	0.167	1.550	2.190	3.014	2.251	0.734
	7.5	6.450	6.500	6.300	**6.417**	**0.104**	6.100	6.350	6.750	**6.400**	**0.328**	9.926	6.235	0.328	5.496	4.841	0.137	0.122	0.147	4.893	3.454	0.151	2.833	2.431
	10	8.350	8.450	8.450	**8.417**	**0.058**	8.750	8.750	8.850	**8.783**	**0.058**	5.235	5.235	4.152	4.874	0.625	0.117	0.122	0.102	4.117	3.954	3.746	3.939	0.186

* these data were excluded from calculation of average and standard deviation (std)

Supplementary Table S3

Raw data for calculating metal removal efficiency (%) and metal uptake capacity (mg/g dry weight) of *Chlamydomonas ulvaensis* Ehr33-6 is presented below. The data includes three replicates of metal concentrations measured by ICP-MS, along with the dry weight of the microalgae recorded at the end of the experiment (after 96 hours) by drying the filtrates in an oven at 60°C for 24 hours. In this study, the concentration of copper (Cu) was higher in the initial media concentrations due to the presence of Cu in the BBM (Bold's Basal Medium). Because Cu is an essential element for photosynthesis, it could not be removed from the media. The Cu concentration in the control medium was measured at 1.66 ± 0.01 mg/L using ICP-MS.

Metal		Initial concentration mg/L					Final metal concentration, mg/L					Removal efficiency, %					Dry weight, mg/L			Uptake capacity, mg/g				
	Nominal	replicates			average	std	replicates			average	std	replicates			average	std	replicates			replicates			Average	std
		1	2	3			1	2	3			1	2	3			1	2	3	1	2	3		
Cu	2	3.555	3.340	3.310	**3.402**	**0.134**	0.555	0.535	0.620	**0.570**	**0.044**	83.684	84.272	81.774	83.244	1.306	0.170	0.180	0.180	16.745	15.926	15.454	16.042	0.653
	5	5.900	5.950	5.900	**5.917**	**0.029**	0.875	0.940	1.020	**0.945**	**0.073**	85.211	84.113	82.761	84.028	1.228	0.170	0.170	0.180	29.657	29.275	27.204	28.712	1.320
	10	10.350	11.100	10.500	**10.650**	**0.397**	1.925	1.755	1.855	**1.845**	**0.085**	81.925	83.521	82.582	82.676	0.802	0.195	0.190	0.190	44.744	46.816	46.289	45.950	1.077
	15	13.900	14.000	13.800	**13.900**	**0.100**	2.990	2.325	2.745	**2.687**	**0.336**	78.489	83.273	80.252	80.671	2.420	0.210	0.195	0.205	51.952	59.359	54.415	55.242	3.772
	20	18.000	17.750	18.000	**17.917**	**0.144**	4.985	4.530	nd	**4.758**	**0.322**	72.177	74.716	nd	79.163	9.982	0.180	0.190	nd	71.843	70.456	nd	71.149	0.980
Cd	0.5	0.391	0.409	0.383	**0.394**	**0.013**	0.356	0.251	0.207	**0.271**	**0.077**	9.606*	36.267	47.440	41.854	7.900	0.19*	0.180	0.230	0.199*	0.794	0.812	0.803	0.013
	2	1.570	1.615	1.585	**1.590**	**0.023**	0.960	0.935	0.895	**0.930**	**0.033**	39.623	41.195	43.711	41.509	2.062	0.165	0.195	0.190	3.818	3.359	3.658	3.612	0.233
	5	3.915	3.720	3.845	**3.827**	**0.099**	2.630	2.785	2.665	**2.693**	**0.081**	31.272	27.221	30.357	29.617	2.124	0.155	0.140	0.135	7.720	7.440	8.605	7.922	0.608
	7.5	5.650	5.500	5.800	**5.650**	**0.150**	3.955	3.955	3.825	**3.912**	**0.075**	30.000	30.000	32.301	30.767	1.328	0.125	0.135	0.120	13.560	12.556	15.208	13.775	1.339
	10	7.050	7.100	7.500	**7.217**	**0.247**	4.840	5.000	4.875	**4.905**	**0.084**	32.933	30.716	32.448	32.032	1.166	0.115	0.105	0.125	20.667	21.111	18.733	20.170	1.264
Cr	0.5	0.418	0.418	0.374	**0.403**	**0.026**	0.080	0.092	0.044	**0.072**	**0.025**	80.748	77.842	89.357	82.649	5.988	0.215	0.200	0.240	1.551	1.607	1.537	1.565	0.037
	2	1.685	1.660	1.685	**1.677**	**0.014**	1.265	1.350	1.225	**1.280**	**0.064**	24.727	19.669	27.107	23.835	3.798	0.200	0.200	0.205	2.078	1.653	2.222	1.984	0.296
	5	4.180	4.220	4.175	**4.192**	**0.025**	3.995	3.755	3.945	**3.898**	**0.127**	13.705	18.889	14.785	15.793	2.735	0.140	0.140	0.115	4.532	6.246	5.952	5.576	0.917
	7.5	6.450	6.500	6.300	**6.417**	**0.104**	5.850	6.300	6.250	**6.133**	**0.247**	13.618	6.973	7.711	9.434	3.642	0.090	0.105	0.095	10.247	4.497	5.497	6.747	3.072
	10	8.350	8.450	8.450	**8.417**	**0.058**	8.350	8.400	8.450	**8.400**	**0.050**	9.567	9.025	8.484	9.025	0.542	0.095	0.095	0.090	9.298	8.772	8.704	8.925	0.325

* these data were excluded from calculation of average and standard deviation (std)

nd – no data points (not measured)

Supplementary Table S4

Raw data for calculating metal removal efficiency (%) and metal uptake capacity (mg/g dry weight) of *Tetradesmus obliquus* Ehr33-9 is presented below. The data includes three replicates of metal concentrations measured by ICP-MS, along with the dry weight of the microalgae recorded at the end of the experiment (after 96 hours) by drying the filtrates in an oven at 60°C for 24 hours. In this study, the concentration of copper (Cu) was higher in the initial media concentrations due to the presence of Cu in the BBM (Bold's Basal Medium). Because Cu is an essential element for photosynthesis, it could not be removed from the media. The Cu concentration in the control medium was measured at 1.66 ± 0.01 mg/L using ICP-MS.

Metal	Initial concentration mg/L						Final metal concentration, mg/L					Removal efficiency, %					Dry weight, mg/L			Uptake capacity, mg/g				
	Nominal	replicates			average	std	replicates			average	std	replicates			average	std	replicates			replicates			Average	std
		1	2	3			1	2	3			1	2	3			1	2	3	1	2	3		
Cu	2	3.555	3.340	3.310	**3.402**	**0.134**	0.585	0.550	0.590	**0.575**	**0.022**	82.803	83.831	82.656	83.097	0.641	0.160	0.195	0.185	17.604	14.624	15.198	15.809	1.581
	5	5.900	5.950	5.900	**5.917**	**0.029**	0.955	1.075	0.830	**0.953**	**0.123**	83.859	81.831	85.972	83.887	2.071	0.180	0.185	0.195	27.565	26.171	26.085	26.607	0.830
	10	10.350	11.100	10.500	**10.650**	**0.397**	0.910	0.835	0.775	**0.840**	**0.068**	91.455	92.160	92.723	92.113	0.635	0.210	0.200	0.215	46.381	49.075	45.930	47.129	1.701
	15	13.900	14.000	13.800	**13.900**	**0.100**	0.655	0.885	1.145	**0.895**	**0.245**	95.288	93.633	91.763	93.561	1.764	0.180	0.195	0.205	73.583	66.744	62.220	67.515	5.721
	20	18.000	17.750	18.000	**17.917**	**0.144**	0.815	0.790	0.945	**0.850**	**0.083**	95.451	95.591	94.726	95.256	0.464	0.190	0.185	0.195	90.009	92.577	87.034	89.873	2.774
Cd	0.5	0.391	0.409	0.383	**0.394**	**0.013**	0.113	0.102	0.088	**0.101**	**0.013**	71.308	74.101	77.782	74.397	3.248	0.200	0.185	0.200	1.404	1.577	1.532	1.504	0.090
	2	1.570	1.615	1.585	**1.590**	**0.023**	0.565	0.545	0.560	**0.557**	**0.010**	64.465	65.723	64.780	64.990	0.655	0.115	0.135	0.115	8.913	7.741	8.957	8.537	0.690
	5	3.915	3.720	3.845	**3.827**	**0.099**	2.005	1.980	2.010	**1.998**	**0.016**	47.605	48.258	47.474	47.779	0.420	0.110	0.090	0.095	16.561	20.519	19.123	18.734	2.007
	7.5	5.650	5.500	5.800	**5.650**	**0.150**	3.170	3.165	2.975	**3.103**	**0.111**	43.894	43.982	47.345	45.074	1.968	0.100	0.110	0.110	24.800	22.591	24.318	23.903	1.162
	10	7.050	7.100	7.500	**7.217**	**0.247**	3.565	3.615	3.795	**3.658**	**0.121**	50.600	49.908	47.413	49.307	1.676	0.120	0.120	0.095	30.431	30.014	36.018	32.154	3.352
Cr	0.5	0.418	0.418	0.374	**0.403**	**0.026**	0.326	0.236	0.333	**0.298**	**0.054**	21.176	42.849	19.360	27.795	13.069	0.195	0.200	0.200	0.448	0.885	0.400	0.578	0.267
	2	1.685	1.660	1.685	**1.677**	**0.014**	1.355	1.370	1.115	**1.280**	**0.143**	19.372	18.479	33.653	23.835	8.514	0.225	0.210	0.265	1.447	1.479	2.134	1.687	0.388
	5	4.180	4.220	4.175	**4.192**	**0.025**	4.125	3.950	3.905	**3.993**	**0.116**	10.896	14.677	15.649	13.741	2.511	0.205	0.175	0.195	2.461	3.883	3.715	3.353	0.777
	7.5	6.450	6.500	6.300	**6.417**	**0.104**	5.900	6.550	6.300	**6.250**	**0.328**	12.879	3.281	6.973	7.711	4.841	0.175	0.160	0.165	4.984	1.389	2.862	3.078	1.807
	10	8.350	8.450	8.450	**8.417**	**0.058**	8.900	8.900	9.050	**8.950**	**0.087**	3.610	3.610	1.986	3.069	0.938	0.145	0.110	0.115	2.299	3.030	1.594	2.308	0.718

* these data were excluded from calculation of average and standard deviation (std)

Supplementary Table S5

Raw data for calculating metal removal efficiency (%) and metal uptake capacity (mg/g dry weight) of *Pseudanabaena catenata* Ehr15-5 is presented below. The data includes three replicates of metal concentrations measured by ICP-MS, along with the dry weight of the microalgae recorded at the end of the experiment (after 96 hours) by drying the filtrates in an oven at 60°C for 24 hours. In this study, the concentration of copper (Cu) was higher in the initial media concentrations due to the presence of Cu in the BBM (Bold's Basal Medium). Because Cu is an essential element for photosynthesis, it could not be removed from the media. The Cu concentration in the control medium was measured at 1.66 ± 0.01 mg/L using ICP-MS.

Metal	Initial concentration mg/L						Final metal concentration, mg/L					Removal efficiency, %					Dry weight, mg/L			Uptake capacity, mg/g				
	Nominal	replicates			average	std	replicates			average	std	replicates			average	std	replicates			replicates			Average	std
		1	2	3			1	2	3			1	2	3			1	2	3	1	2	3		
Cu	0.5	2.150	2.165	2.055	**2.123**	**0.060**	0.261	0.134	0.110	**0.168**	**0.081**	87.732	93.713	94.819	92.088	3.813	0.075	0.070	0.080	24.838	28.426	25.167	26.144	1.984
	2	3.555	3.340	3.310	**3.402**	**0.134**	0.031	0.775	0.493	**0.433**	**0.376**	99.09*	77.217	85.507	81.362	5.862	0.095	0.095	0.095	35.482	27.649	30.618	31.250	3.955
	5	5.900	5.950	5.900	**5.917**	**0.029**	1.865	1.070	1.860	**1.598**	**0.458**	68.479	81.92*	68.563	68.521	0.060	0.100	0.085	0.080	40.517	57.020	50.708	49.415	8.327
	7.5	8.250	8.250	7.550	**8.017**	**0.404**	2.445	1.205	3.055	**2.235**	**0.943**	69.501	84.97*	61.892	65.696	5.380	0.095	0.095	0.095	58.649	71.702	52.228	60.860	9.923
	10	10.350	11.100	10.500	**10.650**	**0.397**	2.445	1.205	3.055	**2.235**	**0.943**	77.042	88.68*	71.315	74.178	4.050	0.115	0.100	0.090	71.348	94.450	84.389	83.396	11.583
Cd	0.5	0.391	0.409	0.383	**0.394**	**0.013**	0.096	0.081	0.091	**0.089**	**0.008**	75.624	79.433	76.894	77.317	1.939	0.075	0.090	0.075	3.971	3.476	4.038	3.828	0.307
	2	1.570	1.615	1.585	**1.590**	**0.023**	2.495	0.765	0.735	**1.332**	**1.008**	-56.92*	51.887	53.774	52.830	1.334	0.065	0.045	0.060	-13.923*	18.333	14.250	16.292	2.887
	5	3.915	3.720	3.845	**3.827**	**0.099**	0.005	1.865	1.905	**1.258**	**1.085**	99.86*	51.263	50.218	50.740	0.739	0.065	0.030	0.060	58.792*	65.389	32.028	48.708	23.590
	7.5	5.650	5.500	5.800	**5.650**	**0.150**	2.355	2.340	2.220	**2.305**	**0.074**	58.319	58.584	60.708	59.204	1.310	0.055	0.065	0.060	59.909	50.923	57.167	56.000	4.605
	10	7.050	7.100	7.500	**7.217**	**0.247**	2.880	2.465	2.760	**2.702**	**0.214**	60.092	65.843	61.755	62.564	2.959	0.035	0.075	0.070	123.905*	63.356	63.667	63.511	0.220
Cr	0.5	0.418	0.418	0.374	**0.403**	**0.026**	0.411	0.408	0.411	**0.410**	**0.002**	0.592	1.197	0.471	0.753	0.389	0.175	0.180	0.185	0.014	0.027	0.011	0.017	0.009
	2	1.685	1.660	1.685	**1.677**	**0.014**	1.600	1.635	1.625	**1.620**	**0.018**	4.793	2.711	3.306	3.603	1.073	0.155	0.185	0.165	0.520	0.246	0.337	0.368	0.139
	5	4.180	4.220	4.175	**4.192**	**0.025**	4.460	4.255	4.255	**4.323**	**0.118**	3.660	8.088	8.088	6.612	2.557	0.170	0.180	0.170	0.997	2.080	2.203	1.760	0.664
	7.5	6.450	6.500	6.300	**6.417**	**0.104**	5.350	6.650	6.550	**6.183**	**0.723**	21.001*	1.805	3.281	2.543	1.044	0.135	0.185	0.160	10.535*	0.661	1.389	1.025	0.515
	10	8.350	8.450	8.450	**8.417**	**0.058**	8.700	8.800	8.750	**8.750**	**0.050**	5.776	4.693	5.235	5.235	0.542	0.130	0.120	0.165	4.103	3.611	2.929	3.548	0.589

* these data were excluded from calculation of average and standard deviation (std)

Supplementary Table S6

Raw data for calculating metal removal efficiency (%) and metal uptake capacity (mg/g dry weight) of *Chlorella vulgaris* SAG211-11b (reference strain) is presented below. The data includes three replicates of metal concentrations measured by ICP-MS, along with the dry weight of the microalgae recorded at the end of the experiment (after 96 hours) by drying the filtrates in an oven at 60°C for 24 hours. In this study, the concentration of copper (Cu) was higher in the initial media concentrations due to the presence of Cu in the BBM (Bold's Basal Medium). Because Cu is an essential element for photosynthesis, it could not be removed from the media. The Cu concentration in the control medium was measured at 1.66 ± 0.01 mg/L using ICP-MS.

Metal	Nominal	Initial concentration mg/L					Final metal concentration, mg/L					Removal efficiency, %					Dry weight, mg/L			Uptake capacity, mg/g				
		replicates			average	std	replicates			average	std	replicates			average	std	replicates			replicates			Average	std
		1	2	3			1	2	3			1	2	3			1	2	3	1	2	3		
Cu	0.5	2.150	2.165	2.055	**2.123**	**0.060**	0.375	0.368	0.363	**0.368**	**0.006**	82.339	82.692	82.928	82.653	0.296	0.135	0.125	0.150	12.951	14.047	11.739	12.912	1.154
	2	3.555	3.340	3.310	**3.402**	**0.134**	0.695	0.700	0.670	**0.688**	**0.016**	79.569	79.422	80.304	79.765	0.472	0.150	0.145	0.140	18.044	18.632	19.512	18.730	0.739
	5	5.900	5.950	5.900	**5.917**	**0.029**	0.875	0.910	1.230	**1.005**	**0.196**	85.211	84.620	79.211	83.014	3.307	0.135	0.130	0.140	37.346	38.513	33.476	36.445	2.636
	7.5	8.250	8.250	7.550	**8.017**	**0.404**	1.260	1.080	1.250	**1.197**	**0.101**	84.283	86.528	84.407	85.073	1.262	0.130	0.145	0.150	51.974	47.839	45.111	48.308	3.456
	10	10.350	11.100	10.500	**10.650**	**0.397**	1.500	1.625	1.775	**1.633**	**0.138**	85.915	84.742	83.333	84.664	1.293	0.120	0.145	0.140	76.250	62.241	63.393	67.295	7.777
Cd	0.5	0.391	0.409	0.383	**0.394**	**0.013**	0.347	0.253	0.227	**0.276**	**0.063**	11.892*	35.760	42.488	39.124	4.758	0.135	0.135	0.125	0.347	1.043	1.339	0.910	0.509
	2	1.570	1.615	1.585	**1.590**	**0.023**	0.775	0.820	0.825	**0.807**	**0.028**	51.258	48.428	48.113	49.266	1.732	0.140	0.125	0.115	5.821	6.160	6.652	6.211	0.418
	5	3.915	3.720	3.845	**3.827**	**0.099**	1.895	1.970	1.970	**1.945**	**0.043**	50.479	48.519	48.519	49.172	1.132	0.140	0.140	0.120	13.798	13.262	15.472	14.177	1.153
	7.5	5.650	5.500	5.800	**5.650**	**0.150**	2.940	3.045	3.040	**3.008**	**0.059**	47.965	46.106	46.195	46.755	1.048	0.115	0.120	0.110	23.565	21.708	23.727	23.000	1.122
	10	7.050	7.100	7.500	**7.217**	**0.247**	3.585	3.625	4.015	**3.742**	**0.238**	50.323	49.769	44.365	48.152	3.292	0.120	0.115	0.105	30.264	31.232	30.492	30.663	0.506
Cr	0.5	0.418	0.418	0.374	**0.403**	**0.026**	0.304	0.412	0.399	**0.371**	**0.059**	26.503*	0.350	3.377	1.863	2.140	0.140	0.135	0.130	0.782*	0.011	0.107	0.059	0.068
	2	1.685	1.660	1.685	**1.677**	**0.014**	1.605	1.600	1.600	**1.602**	**0.003**	4.496	4.793	4.793	4.694	0.172	0.120	0.110	0.120	0.630	0.732	0.671	0.678	0.052
	5	4.180	4.220	4.175	**4.192**	**0.025**	4.135	4.260	4.520	**4.305**	**0.196**	10.680	7.980	2.364	7.008	4.243	0.085	0.085	0.095	5.817	4.346	1.152	3.772	2.385
	7.5	6.450	6.500	6.300	**6.417**	**0.104**	6.500	6.650	6.700	**6.617**	**0.104**	4.020	1.805	1.066	2.297	1.537	0.055	0.065	0.070	4.949	1.880	1.032	2.621	2.061
	10	8.350	8.450	8.450	**8.417**	**0.058**	8.700	8.850	8.850	**8.800**	**0.087**	5.776	4.152	4.152	4.693	0.938	0.050	0.055	0.070	10.667	6.970	5.476	7.704	2.672

* these data were excluded from calculation of average and standard deviation (std)

Supplementary Table S7.

The overall one-way ANOVA test results of heavy metal removal rate and removal by dry biomass from each algal isolates.

Isolate	Metal	Removal	DF	Sum of Squares	Mean Square	F-value	p-value
RG1-4	Cu	Removal rate	4	627.3804	156.8451	19.45864	1.04E-04
		Removal by dry biomass	4	13370.91	3342.727	105.1407	<0.0001
	Cd	Removal rate	4	598.2193	149.5548	30.95108	<0.0001
		Removal by dry biomass	4	350.3152	87.57881	139.8875	<0.0001
	Cr(VI)	Removal rate	4	529.6695	132.4174	27.11636	<0.0001
		Removal by dry biomass	4	49.0226	12.25565	6.08866	0.0095
Ehr31-1	Cu	Removal rate	4	177.7409	44.43523	27.08312	<0.0001
		Removal by dry biomass	4	18933.66	4733.415	30.92279	<0.0001
	Cd	Removal rate	4	1357.145	339.2863	14.54636	3.57E-04
		Removal by dry biomass	4	17355.24	4338.809	389.2911	<0.0001
	Cr(VI)	Removal rate	4	14246.5	3561.625	396.0322	<0.0001
		Removal by dry biomass	4	47.31671	11.82918	8.9276	0.00246
Ehr33-6	Cu	Removal rate	4	47.76629	11.94157	0.54602	0.70622
		Removal by dry biomass	4	4819.057	1204.764	300.5646	<0.0001
	Cd	Removal rate	4	386.7201	96.68002	10.09585	0.0022
		Removal by dry biomass	4	744.3793	186.0948	236.073	<0.0001
	Cr(VI)	Removal rate	4	13885.62	3471.404	227.2832	<0.0001
		Removal by dry biomass	4	13.54836	3.38709	1.86029	0.19414
Ehr33-9	Cu	Removal rate	4	386.7627	96.69067	57.36662	<0.0001
		Removal by dry biomass	4	10857.87	2714.469	291.8422	<0.0001
	Cd	Removal rate	4	1951.642	487.9105	136.8023	<0.0001
		Removal by dry biomass	4	1776.106	444.0264	129.8218	<0.0001
	Cr(VI)	Removal rate	4	1124.825	281.2063	31.3137	<0.0001
		Removal by dry biomass	4	72.89965	18.22491	15.28926	4.78E-04
Ehr15-5	Cu	Removal rate	4	1129.228	282.307	15.56867	0.00254
		Removal by dry biomass	4	6462.056	1615.514	25.1204	<0.0001
	Cd	Removal rate	4	1149.771	287.4426	74.67758	<0.0001
		Removal by dry biomass	4	6916.885	1729.221	19.92608	6.31E-04
	Cr(VI)	Removal rate	4	101.3798	25.34495	11.12928	0.00156
		Removal by dry biomass	4	14.10626	3.52657	16.46837	3.59E-04
SAG211-11b	Cu	Removal rate	4	52.93498	13.23375	4.56072	0.02354
		Removal by dry biomass	4	5878.953	1469.738	90.44709	<0.0001
	Cd	Removal rate	4	157.7877	39.44692	6.44701	0.00988
		Removal by dry biomass	4	1528.013	382.0033	565.4176	<0.0001
	Cr(VI)	Removal rate	4	148.7838	37.19594	5.96139	0.01259
		Removal by dry biomass	4	96.52007	24.13002	14.42419	5.96E-04

Supplementary Table S8.

EC50 values for other algal strains in response to copper (Cu).

Organism and reference	EC50 value, mg/L
Isochrysis galbana[1]	0.0058
Rhodomonas salina[1]	0.048
Chaetoceros sp.[1]	0.088
Cyanobium sp. NIES-981[2]	0.09
Microcystis aeruginosa FACHB-469[3]	0.093
Chlorella vulgaris[4]	0.1
Nannochloropsis gaditana[1]	0.137
Tetraselmus chuii[1]	0.33
Bathycoccus prasinos NIES-2670[2]	0.37
Chlorolobion braunii[5]	2.13
Chlamydomonas reinhardtii SAG 11-32b[6]	3.04
Emiliania huxleyi NIES-1310[2]	5.2
Tetradesmus obliquus[7]	5.838
Chlamydomonas reinhardtii[8]	6.35
Tetraselmus chuii[9]	6.44
Scenedesmus incrassatulus[10]	6.57
Synechococcus sp.[11]	0.27
Microcystis aeruginosa[11]	0.0635
Dolichospermum flosaquae[11]	0.091
Scenedesmus quadricauda[11]	19.969
Chlorella kessleri[11]	16.418
Chlamydomonas reinhardtii[11]	23.304
Cyclotella meneghiniana[11]	0.36
Asterionella formosa[11]	2.69
Navicula pelliculosa[11]	0.269
Tetradesmus obliquus[12]	0.05
Chlorella pyrenoidosa[12]	0.068
Closterium lunula[12]	0.2
Navicula permitis[13]	29.41
Dunaliella salina[14]	18.14
Pseudokirchneriella subcapitata[15]	0.048
Phaeodactylum tricornutum[16]	1.205

Supplementary Table S9.

EC50 values for other algal strains in response to cadmium (Cd).

Organism and reference	EC50 value, mg/L
Microcystis aeruginosa[17]	0.001
Tetradesmus obliquus[18]	0.005
Scenedesmus quadricauda[19]	0.008
Microcoleus vaginatus[18]	0.015

Ankistrodesmus falcatus[18]	0.019
Selenastrum capricornutum[18]	0.019
Chlamydomonas reinhardtii[20]	0.021
Tetradesmus obliquus[7]	0.0594
Pseudokirchneriella subcapitata[17]	0.067
Microcystis aeruginosa FACHB-469[3]	0.1
Chlorolobion braunii[5]	0.18
Non-flocullating *Chlorella vulgaris* CNW11[21]	1.7
Flocullating *Chlorella vulgaris* JSC-7[21]	2.1
Nannochloropsis oculata[22]	4.97
Tetraselmus chuii[9]	5.44
Chlamydomonas reinhardtii CC-125[23]	6.29
Scenedesmus incrassatulus[10]	7.36
Chlamydomonas reinhardtii – negatively phototactic mutant (agg1)[23]	8.68
Chlamydomonas reinhardtii SAG 11-32b[6]	11.1
Chlamydomonas. reinhardtii cw15-325[24]	11.24
Chlamydomonas reinhardtii CC-125[25]	12.48
Chlamydomonas reinhardtii CW15mt- cell wall less strain[26]	16.86
Scenedesmus quadricauda[27]	20
Phormidium ambigum[27]	40
Pseudochlorococcum typicum[27]	40
Navicula permitis[13]	7.86
Raphidocelis subcapitata[28]	0.08
Dunaliella salina[14]	3.32
Pseudokirchneriella subcapitata[15]	0.018
Phaeodactylum tricornutum[16]	2.494
Tetradesmus obliquus[29]	5.11
Chlorella pyrenoidosa[29]	5.12
Selenastrum capricornutum[29]	4.85
Tetradesmus obliquus[30]	0.41

Supplementary Table S10.

EC50 values for other algal strains in response to chromium (Cr(VI)).

Organism and reference	EC50 value, mg/L
Scenedesmus quadricauda[19]	0.54
Microcystis aeruginosa[17]	0.58
Pseudokirchneriella subcapitata[17]	1.04
Scenedesmus incrassatulus[10]	2.09
Chlamydomonas reinhardtii SAG 11-32b[6]	2.19
Microcystis aeruginosa FACHB-469[3]	2.98
Phaeodactylum tricornutum CCY0033[31]	4.3
Tetradesmus obliquus[7]	4.526
Scenedesmus dimorphus[32]	5
Chlorella sp.[32]	5

Navicula pelliculosa CCMP543[31]	8.2
Planothidium lanceolatum[33]	8.7
Navivula subminuscula[34]	9.2
Craticula subminuscula[35]	15.12
Parachlorella kessleri Bh-2[36]	30
Pseudokirchneriella subcapitata[15]	0.2
Phaeodactylum tricornutum[16]	15.378

References:

1. Debelius, B., Forja, J. M., DelValls, Á. & Lubián, L. M. Toxicity and bioaccumulation of copper and lead in five marine microalgae. *Ecotoxicology and Environmental Safety* **72**, 1503–1513 (2009).
2. Ota, S. *et al.* Differential heavy metal sensitivity in seven algal species from the NIES culture collection based on delayed fluorescence assays. *Phycological Research* **68**, 41–49 (2020).
3. Gao, C., Gao, L., Duan, P., Wu, H. & Li, M. Evaluating combined toxicity of binary heavy metals to the cyanobacterium *Microcystis*: A theoretical non-linear combined toxicity assessment method. *Ecotoxicology and Environmental Safety* **187**, 109809 (2020).
4. Juneau, P., El Berdey, A. & Popovic, R. PAM fluorometry in the determination of the sensitivity of Chlorella vulgaris, Selenastrum capricornutum, and Chlamydomonas reinhardtii to copper. *Arch Environ Contam Toxicol* **42**, 155–164 (2002).
5. Echeveste, P., Silva, J. C. & Lombardi, A. T. Cu and Cd affect distinctly the physiology of a cosmopolitan tropical freshwater phytoplankton. *Ecotoxicology and Environmental Safety* **143**, 228–235 (2017).
6. Nowicka, B., Pluciński, B., Kuczyńska, P. & Kruk, J. Physiological characterization of *Chlamydomonas reinhardtii* acclimated to chronic stress induced by Ag, Cd, Cr, Cu and Hg ions. *Ecotoxicology and Environmental Safety* **130**, 133–145 (2016).
7. Danouche, M., El Ghachtouli, N., El Baouchi, A. & El Arroussi, H. Heavy metals phycoremediation using tolerant green microalgae: Enzymatic and non-enzymatic antioxidant systems for the management of oxidative stress. *Journal of Environmental Chemical Engineering* **8**, 104460 (2020).
8. Ibuot, A., Dean, A. P., McIntosh, O. A. & Pittman, J. K. Metal bioremediation by *CrMTP4* over-expressing *Chlamydomonas reinhardtii* in comparison to natural wastewater-tolerant microalgae strains. *Algal Research* **24**, 89–96 (2017).

9. Cordero, J., Guevara, M., Morales, E. & Lodeiros, C. Effect of heavy metals on the growth of tropical microalga Tetrasermis chuii (Prasinophyceae). *Revista de biología tropical* **53**, 325–30 (2005).

10. Pena-Castro, J. M. Interaccion de los metales pesados Cd, Cu y Cr con la microalga Scenedesmus incrassatulus: importancia biologica y evaluacion de su capacidad de remocion. (Cinvestav-IPN-Mexico., 2002).

11. Mehdizadeh Allaf, M., Erratt, K. J. & Peerhossaini, H. Comparative assessment of algaecide performance on freshwater phytoplankton: Understanding differential sensitivities to frame cyanobacteria management. *Water Research* **234**, 119811 (2023).

12. Yan, H. & Pan, G. Toxicity and bioaccumulation of copper in three green microalgal species. *Chemosphere* **49**, 471–476 (2002).

13. Elhamji, S. *et al.* Addressing the challenge of heavy metal contamination in aquatic environment: Harnessing the bioremediation potential of 'Navicula permitis' diatom for wastewater treatment. *Biomass and Bioenergy* **197**, 107776 (2025).

14. Gao, M., Ling, N., Tian, H., Guo, C. & Wang, Q. Toxicity, physiological response, and biosorption mechanism of Dunaliella salina to copper, lead, and cadmium. *Front Microbiol* **15**, 1374275 (2024).

15. Al-Hasawi, Z. M., Abdel-Hamid, M. I., Almutairi, A. W. & Touliabah, H. E. Response of Pseudokirchneriella subcapitata in Free and Alginate Immobilized Cells to Heavy Metals Toxicity. *Molecules* **25**, 2847 (2020).

16. Mercado, B., Valero, N., Roca-Pérez, L., Bernabeu-Berni, E. & Andreu-Sánchez, O. Investigation of Metal Toxicity on Microalgae Phaeodactylum tricornutum, Hipersaline Zooplankter Artemia salina, and Jellyfish Aurelia aurita. *Toxics* **11**, 716 (2023).

17. Rodgher, S., Espíndola, E. L. G., Simões, F. C. F. & Tonietto, A. E. Cadmium and Chromium Toxicity to Pseudokirchneriella subcapitata and Microcystis aeruginosa. *Braz. arch. biol. technol.* **55**, 161–169 (2012).

18. Vocke, R. W. Growth responses of selected freshwater algae to trace elements and scrubber ash slurry by coal-fired power plants. (Iowa State University, Ames, Iowa, 1978).

19. Fargašsová, A. Comparative toxicity of five metals on various biological subjects. *Bull. Environ. Contam. Toxicol.* **53**, 317–324 (1994).

20. Islam, Md. S., Sazawa, K., Hata, N., Sugawara, K. & Kuramitz, H. Determination of heavy metal toxicity by using a micro-droplet hydrodynamic voltammetry for microalgal bioassay based on alkaline phosphatase. *Chemosphere* **188**, 337–344 (2017).

21. Alam, Md. A. *et al.* Enhanced removal of Zn2+ or Cd2+ by the flocculating Chlorella vulgaris JSC-7. *Journal of Hazardous Materials* **289**, 38–45 (2015).

22. Zamani-Ahmadmahmoodi, R., Malekabadi, M. B., Rahimi, R. & Johari, S. A. Aquatic pollution caused by mercury, lead, and cadmium affects cell growth and pigment content of marine microalga, Nannochloropsis oculata. *Environ Monit Assess* **192**, 330 (2020).

23. Yu, Z., Zhang, T., Hao, R. & Zhu, Y. Sensitivity of Chlamydomonas reinhardtii to cadmium stress is associated with phototaxis. *Environ. Sci.: Processes Impacts* **21**, 1011–1020 (2019).

24. Zalutskaya, Z., Ostroukhova, M. & Ermilova, E. The *Chlamydomonas* alternative oxidase 1 is regulated by cadmium stress: New insights into control of expression. *Environmental and Experimental Botany* **130**, 133–140 (2016).

25. Li, C. *et al.* Contrasting detoxification mechanisms of Chlamydomonas reinhardtii under Cd and Pb stress. *Chemosphere* **274**, 129771 (2021).

26. Gillet, S., Decottignies, P., Chardonnet, S. & Le Maréchal, P. Cadmium response and redoxin targets in Chlamydomonas reinhardtii: a proteomic approach. *Photosynth Res* **89**, 201–211 (2006).

27. Shanab, S., Essa, A. & Shalaby, E. Bioremoval capacity of three heavy metals by some microalgae species (Egyptian Isolates). *Plant Signaling & Behavior* **7**, 392–399 (2012).

28. dos Reis, L. L. *et al.* Effects of cadmium and cobalt mixtures on growth and photosynthesis of *Raphidocelis subcapitata* (Chlorophyceae). *Aquatic Toxicology* **244**, 106077 (2022).

29. Mo, L. *et al.* Time-Dependent Toxicity and Health Effects Mechanism of Cadmium to Three Green Algae. *Int J Environ Res Public Health* **19**, 10974 (2022).

30. Xu, P. *et al.* Cadmium-Induced Physiological Responses, Biosorption and Bioaccumulation in Scenedesmus obliquus. *Toxics* **12**, 262 (2024).

31. Hedayatkhah, A., Cretoiu, M. S., Emtiazi, G., Stal, L. J. & Bolhuis, H. Bioremediation of chromium contaminated water by diatoms with concomitant lipid accumulation for biofuel production. *Journal of Environmental Management* **227**, 313–320 (2018).

32. Nath, A., tiwari, P. K., Rai, A. K. & Sundaram, S. Microalgal consortia differentially modulate progressive adsorption of hexavalent chromium. *Physiol Mol Biol Plants* **23**, 269–280 (2017).

33. Karim, S., Cherifi, O. & Bertrand. Toxicity and biosorption of chromium from aqueous solutions by the diatom Planothidium lanceolatum (Brébisson) Lange-Bertalot. *American Journal of Scientific and Industrial Research* **3**, 27–38 (2012).

34. Cherifi, O., Sbihi, K., Bertrand, M. & Cherifi, K. The siliceous microalga Navicula subminuscula (Manguin) as a biomaterial for removing metals from tannery effluents: a laboratory study. *Journal of Materials and Environmental Sciences* **8**, 884–893 (2017).

35. Karim, S. *et al.* Biosorption of Hexavalent Chromium by Freshwater Microalgae Craticula subminuscula from Aqueous Solutions. *Sustainability* **16**, 918 (2024).

36. Bauenova, M. O. *et al.* Potential of microalgae Parachlorella kessleri Bh-2 as bioremediation agent of heavy metals cadmium and chromium. *Algal Research* **59**, 102463 (2021).
